# Defining fitness in an uncertain world

**DOI:** 10.1007/s00285-017-1164-z

**Published:** 2017-07-29

**Authors:** Paul Crewe, Richard Gratwick, Alan Grafen

**Affiliations:** 1 St John’s College, University of Oxford, Oxford, OX1 3JP UK; 2 Zoology Department, University of Oxford, Oxford, OX1 3PS UK

**Keywords:** Reproductive value, Class-distribution stochasticity, Stochastic environment, Price equation, Formal darwinism, Fundamental theorem of natural selection, 60J99, 92D15

## Abstract

The recently elucidated definition of fitness employed by Fisher in his fundamental theorem of natural selection is combined with reproductive values as appropriately defined in the context of both random environments and continuing fluctuations in the distribution over classes in a class-structured population. We obtain astonishingly simple results, generalisations of the Price Equation and the fundamental theorem, that show natural selection acting only through the arithmetic expectation of fitness over all uncertainties, in contrast to previous studies with fluctuating demography, in which natural selection looks rather complicated. Furthermore, our setting permits each class to have its characteristic ploidy, thus covering haploidy, diploidy and haplodiploidy at the same time; and allows arbitrary classes, including continuous variables such as condition. The simplicity is achieved by focussing just on the effects of natural selection on genotype frequencies: while other causes are present in the model, and the effect of natural selection is assessed in their presence, these causes will have their own further effects on genoytpe frequencies that are not assessed here. Also, Fisher’s uses of reproductive value are shown to have two ambivalences, and a new axiomatic foundation for reproductive value is endorsed. The results continue the formal darwinism project, and extend support for the individual-as-maximising-agent analogy to finite populations with random environments and fluctuating class-distributions. The model may also lead to improved ways to measure fitness in real populations.

## Introduction

The individual-as-maximising-agent analogy is a central principle in many sub-disciplines of biology, but in other sub-disciplines is regarded as inaccurate and misleading. Here, we study this question in structured populations with stochastic demography, that is, with continuing fluctuations in class distribution. Such populations have previously been discussed and studied by Tuljapurkar (1989, 1990) from a demographic point of view, and in population genetic terms by Rousset (2004); but the results presented here, in pursuit of fitness-maximising ideas, are new and astonishingly simple by comparison.

The main novel results here are a generalisation of the Price Equation (Price 1970, 1972) and of Fisher (1930)’s fundamental theorem. These do not immediately map on to more traditional conclusions and their relevance is still being discussed in other applications (e.g. Ewens 2004; Edwards 2014; Grafen 2015a; Ewens and Lessard 2015), as well as in the ‘Reproductive value’ section of the Discussion below. The simplicity of the formulae is a powerful additional reason to seek useful interpretations in the context of fluctuating demography. The formulae are rigorously proved, but unfortunately it has not been possible here to set out definitive links to, for example, ESS theory and related ideas, even though that is a very desirable next step. The existence of an individual fitness with very attractive properties will offer hope, however.

Tuljapurkar (1989, section 4.4), Rousset (2004, eqn 10.15) and Rousset and Ronce (2004, eqn 8) each define reproductive value under fluctuating demography, and the simple results here follow from taking an essentially similar definition of reproductive value, though technically different because of the setting, and using it to define ‘fitness’ following Fisher (1930)’s definition, as recently clarified by Grafen (2015a). Conceptually here, reproductive value is the fraction of a long distant gene pool whose ancestral paths pass through an individual or a class of individuals today—see the Discussion for alternatives.

The work of Rousset (2004) and colleagues (notably including Rousset and Ronce 2004; Lehmann et al. 2006; Lehmann and Rousset 2014b), deals with the population genetics of fluctuating demography in great depth, and uses essentially the same definition of reproductive value as the present paper. The difference of approach of the present paper has been to pursue a very general model, so general that it lacks dynamic sufficiency, to study connections with Darwinian design, through focussing on individuals and a particular definition of individual fitness. The costs of losing dynamic sufficiency need to be balanced against the advantages of the simple and general outcomes proved here.

The technical definitions of reproductive value and fitness, with all their apparatus, are complicated: now, we provide a simple exposition of the simple central idea for defining fitness from reproductive value. We let the class of an individual *i* this year be $x_i$. The definition of reproductive value attributes to each individual *i*, in each year, a real number that is its reproductive value, which depends only on its class, and we notate the reproductive value assigned in this way as $v_{x_i}$. We follow the lead of Williams (1966) in constructing for an individual *i*, regarded as a parent, a quantity here called the ‘Williams’ reproductive value’ denoted $W_i$, which is the sum over its offspring and its own surviving self of its share of reproductive value from those descendants. In the case of diploid offspring, only half of the offspring’s reproductive value belongs to each parent. In the case of the surviving self, the contribution of reproductive value must be devalued by the chance of survival, and note that the class may change. Specialising to age classes, an individual of age 10 who has a chance 0.9 of surviving with a class-based expected reproductive value next year of $v_{11}$, and has 3 offspring with a class-based expected reproductive value of $v_0$, will have a Williams’ reproductive value of

$$\begin{aligned} W_i = 0.9 v_{11} + (3/2) v_0. \end{aligned}$$

$W_i$ may differ from the reproductive value expected for an age 10 individual because not all age 10 individuals are the same. We are particularly interested in how these deviations from expected are associated with genotypes, and this is what the Price Equation and fundamental theorem work with. To define fitness $F_i$ for individual *i*, we combine the Williams’ reproductive value with the class-predicted reproductive value as follows,

1 $$\begin{aligned} F_i = \frac{W_i - v_{x_i}}{v_{x_i}}, \end{aligned}$$

and this formula is simply Fisher’s definition transferred to a discrete-time setting (Grafen 2015a, eqn 39).

This relativising of Williams’ reproductive value to the class-predicted reproductive value provides a concept of fitness with a number of technically convenient properties, that together permit the generalisations of the Price Equation and fundamental theorem in which fitness appears only as an arithmetic expectation over relevant uncertainties. It is mathematically inevitable that, when dealing with fluctuating demographic structure and population size, approaches without this definition or some affine equivalent would bring in higher moments of their different version of fitness (or reproductive success or equivalent) in finding equations for the expectation (over that uncertainty) of gene frequency change. The linearity and expectations in those two main results make a very strong case for the naturalness of this extension of Fisher’s definition of fitness to populations with fluctuating demography. The division by reproductive value looks very natural if we accept the dictum of Fisher (1930, p. 36) that all population means should be calculated with reproductive value weighting, and note that the Williams’ reproductive value essentially counts expected number of gene copies. The first thing the weighting does is to cancel with the divisor of fitness, so that we are summing expected number of gene copies, as we need to do.

The concept of fitness has further significant properties suitable for a maximand in the ‘individual-as-maximising-agent’ analogy. It is not a single ‘tombstone’ measure for an individual, but is defined in each year, and in each year is based entirely on future reproduction. It equals zero for an individual who performs as expected for an individual in that class, and equals one for an individual who performs twice as well. Fitness does not measure an absolute contribution to future gene pools (reproductive value does measure an absolute *expected* contribution), but rather how well an individual performs relative to what is expected for its class. Fitness is defined for a population with class-based ploidy, thus including in the same framework haploidy, diploidy and haplodiploidy, and also for arbitrary classes that may include age, but may also incorporate continuous variables such as condition even though the population is finite.

Thus, the results obtained suggest that fitness-maximisation ideas can be extended to a very complex stochastic setting, but their surprising simplicity also suggests that they deserve attention from demographers and population geneticists. Metz et al. (1992) began to define individual fitness within a population genetic context in a very general way. A major question is what a Price Equation and fundamental theorem can contribute to those areas, but one possible prize is being able to draw conclusions about natural selection without having to tackle the complexities of Lyapunov exponents, the approach indicated by Tuljapurkar (1989). The simplicity of the results may inform how selection is best measured empirically, in sophisticated work such as Coulson et al. (2010) that deals with class-structured populations. In this informal preview of the results, we have omitted many central details, which must be fully specified in the sequel. We hope this introduction provides enough motivation for further reading.

## Preliminaries

We first define our basic full model of a class-structured population with environmental and class-distribution stochasticity. This allows the construction of a ‘neutral stochastic process’ that assumes phenotypic uniformity, and then of a ‘Taylor switch stochastic process’. The neutral process will be used to define the key concept of reproductive value, through the construction of a transition operator on a relevant product space. Reproductive value will be one central ingredient of the definition of ‘fitness’. The Taylor switch process takes its initial conditions from the ergodic distribution of the neutral process, and allows genotype and class to affect phenotypes in the first year, but thereafter reverts to the same uniform phenotype as the first process. That special first year, over which we study evolution, is called the ‘Taylor year’.

The idea of defining reproductive value in some ‘null situation’, and then applying it in a ‘non-null situation’, is a technique explained and employed by Taylor (1990, 1996) and later used extensively, and which we will call the ‘Taylor switch’. Taylor’s null situation had a fixed asymptotic class distribution and constant environment, but we have fluctuating demography and varying environments, and so our reproductive value depends not just on class but also on environment and the population class distribution. Taylor’s genetic switch is to have a mutant allele, which is rare and has its effect in only one year, thus his ‘null’ population is phenotypically homogeneous, and genetically homogeneous apart from a rare allele at a single locus, and he looks for an increase in the (reproductive-value-weighted) frequency of that allele. Here, the genetic switch is that an arbitrary genotype-phenotype map is permitted for one year, and our ‘null’ population is again phenotypically homogeneous, but genetically heterogeneous (maintained by recurrent mutation) at an arbitrary number of loci with arbitrary linkage patterns. Our Price Equation gives a formula for the expected change in the frequency of any one allele (and, as the formula is the same for each allele and is linear, of an arbitrary weighted sum of allele frequencies). The extra complexities make a formalisation worthwhile.

Class plays a major role in the model. Class must not be determined by an individual’s genotype, though a parent’s genotype may affect the class of its offspring, including of its own surviving self. In positive terms, it must allow a dynamically sufficient model of class demography in any population in which genotypic variation has no influence on phenotypes. One candidate trait is sex in species with sex chromosomes, when we are studying evolution at autosomes. Another is individual condition. The heritability of height makes it at first sight an unlikely candidate, because it depends on genotype, but the heritability may be due to mutation-selection balance around an equilibrium whose position would be indicated by regarding height as a class.

The dynamic suffiency required here for class structure stands in contrast to the very weak assumptions made about genetics in our model, which imply that the genetic system is not characterised in a dynamically sufficient way. This restricts the kinds of results that can be obtained, but it allows all the conclusions that can be drawn to be very general, as they do not depend on mating system, linkage patterns, linkage disequilibrium, epistasis, dominance and so on. The generalities ascribed to the Price Equation and to the fundamental theorem of natural selection have the same source. In earlier days in population genetics (e.g. Lewontin 1974), dynamic sufficiency was important because erroneous conclusions were being drawn through making convenient but false assumptions. The advantages and disadvantages today of dynamic sufficiency, in models using genetics to study phenotypes, are discussed, with generally opposite conclusions, by Grafen (2008) and Lehmann and Rousset (2014b).

### Basic model

The notation in the introduction was used just to sketch the definition of fitness. Here, we begin again more formally. Consider a finite population in discrete time with overlapping generations, in which each individual belongs to a single class from a (possibly infinite) measure space of classes *X*, and also possesses a genotype from the finite set $\mathcal {G}$. We study the evolution of the population state with an emphasis on the role of individuals. In a given year, the population considered as parents will be described by finite arrays whose *i*th elements describe the class and genotype of the *i*th individual. The number of elements of each of these arrays is the population size, which will vary from year to year. Formally, the array $\mathbf {x}$ denoting the class of each member of the population belongs to $X^k$ if the population is of size *k*, and so unconditionally belongs to $\tilde{X}=\bigcup _{k=1,2\ldots } X^k$. We note that $\tilde{X}$ is the union of a countable number of measure spaces, and so itself forms a measure space with an appropriate sigma algebra (Schechter 1997). Similarly, the array $\mathbf {g}$ denoting the genotype of each member of the population belongs to the measure space $\tilde{\mathcal {G}} = \bigcup _{k=1,2\ldots } \mathcal {G}^k$. Thus the population in year *t* is described by arrays $(\mathbf {x}^{(t)}, \mathbf {g}^{(t)})$ representing the class and genotype of each individual, with typical elements $x^{(t)}_i$ and $g^{(t)}_i$. The environment will be modelled as a Markov process, so that the environment $e'$ in the next year will take values in the measure space $(E,\varSigma )$, according to the measure $\varPi (e,\cdot )$, where $e$ is the current environment. The environment in year *t* will be written as $e^{(t)}$. In order to help understanding, we use $x'$ in place of *x* and $g'$ in place of *g* to index the sets *X* and $\mathcal {G}$ when the individuals referred to are in the offspring year.

Since offspring may be spread over different classes and genotypes, we write the reproductive output of an individual *i* as an array $\mathbf {w}^i = (w^i_{x',g'})_{x'\in X, g'\in \mathcal {G}}$, where $w^i_{x',g'}$ is the number of whole offspring of *i* in class $x'$ with genotype $g'$, *per haploid set of the parent*, weighted by the parent’s genetic contribution. Thus if a diploid parent *i* contributes exactly one haploid gamete to a diploid offspring in class $x'$ with genotype $g'$, we would have $w_{x',g'}^i = 1/4$, because the parent’s genetic contribution to that offspring was 1 / 2, and that half was distributed between the parent’s two haploid sets. As the population is finite, only a finite number of class-genotype combinations have non-zero elements of $\mathbf {w}^i$.

The array of the number of individuals in each class and genotype in the next year is just the ploidy-weighted sum of the offspring arrays of the parents

$$\begin{aligned} \sum _{i\in I} L_i \cdot \mathbf {w}^i = \bigg ( \sum _{i\in I} L_i \cdot w_{x',g'}^i \bigg )_{x'\in X, g'\in \mathcal {G}}, \end{aligned}$$

where $L_i$ is the ploidy of *i*. As the framework is stochastic, the reproductive output $\mathbf {w}^i$ of each individual *i* is an array-valued random variable, whose distribution depends on $( e , \mathbf {x})$, on a probability space $(\varOmega , \mathscr {F}, \mathbb {P})$. Thus to understand the evolution of the process for the class and genotype arrays $(\mathbf {x}, \mathbf {g})$ we need to understand the joint distribution of the offspring functions as the population and environment fluctuate.

#### Remark 1

(i) Throughout this paper ploidy will always be the same for all members of any given class, with the ploidy of class *x* written $L_x$. For example in haplodiploid insects, $L_{\text {Males}} = 1$ and $L_{\text {Females}} = 2$.
(ii) In the common situation in which a parent contributes one haploid set to an offspring, the contribution is measured as one over the offspring ploidy (the share of the offspring that belongs to this parent rather than its other parents) times one over the parent’s ploidy (because the contribution is measured per parental ploidy). Hence, in this case if a parent contributes to just one offspring of class $x'$ with genotype $g'$, then
  $$\begin{aligned} w^\text {Parent}_{x',g'} = \frac{1}{L_{x'} L_{\text {Parent}}}. \end{aligned}$$
(iii) Overlapping generations are handled in the usual way by counting an individual’s survival to the next year as an offspring, all of whose genes come from the individual itself (and, if the model is age structured, in the next age class) (Taylor 1990). Thus, if its own surviving self is the individual’s only “offspring” in class $x'$ with genotype $g'$, then parent and offspring ploidies will usually be equal, and the ploidy rules lead to
  $$\begin{aligned} w^\text {Parent}_{x',g'} = \frac{1}{L_{\text {Parent}}} = \frac{1}{L_{x'}}. \end{aligned}$$
(iv) Throughout the paper, bold lowercase Roman letters such as $\mathbf {u}, \mathbf {v}$ and $\mathbf {w}$ will be arrays, while their elements will be written $u_i, v_i$ and $w^i_{x',g'}$. A year superscript will be added in parentheses where needed.
(v) It is convenient to explain here why the framework applies to a set of loci that all belong to one coreplicon (Cosmides and Tooby 1981), but not when the loci are spread over more than one coreplicon. Suppose a diploid mated pair under XY sex-determination have two daughters and one son, that the classes are $\{\text {female},\text {male}\}$, and for simplicity assume there is only one genotype, $g'$. Then at autosomes, $L_\text {mother}=L_\text {father}=2$, and
  2 $$\begin{aligned} w^\text {mother}_{\text {female},g'}&= 1/2&w^\text {{father}}_{\text {female},g'}&= 1/2 \end{aligned}$$
  3 $$\begin{aligned} w^{\text {mother}}_{\text {male},g'}&= 1/4&w^\text {father}_{\text {male},g'}&= 1/4, \end{aligned}$$
  whereas for the *X*-chromosomes, $L_\text {mother}=2$, $L_\text {father}=1$, and
  4 $$\begin{aligned} w^\text {mother}_{\text {female},g'}&= 1/2&w^\text {{father}}_{\text {female},g'}&= 1 \end{aligned}$$
  5 $$\begin{aligned} w^\text {mother}_{\text {male},g'}&= 1/2&w^{\text {father}}_{\text {male},g'}&= 0 . \end{aligned}$$
  The values for *Y*-chromosomes and for mitochondria are each different again. Thus, when a vertebrate is regarded as having 3 offspring in a Price Equation or a fundamental theorem, we must have already taken a view on whether we are discussing an autosomal locus, or a locus on some other coreplicon. This means that Price (1970)’s equation is restricted to diploids and to autosomal loci; and that the discussion of Ewens (2004, pp. 64–67) of Fisher’s fundamental theorem, framed explicitly in terms of diploidy, is restricted to autosomal loci by the repeated assumption that each individual has two alleles at each locus in question. These points are not controversial, and are made simply to highlight an assumption that is not always noticed or understood. Thus, the most basic data depends on which coreplicon a locus belongs to, and so our analysis will assume that all loci are restricted to just one coreplicon. There are many discussions of the genetic conflicts that arise as consequence of the opposing effects of natural selection in different coreplicons (starting with Cosmides and Tooby 1981).

The philosophy of our approach is that the reproduction of individuals should be thought of as random (as in Rice 2008), and that it is the probability distribution of reproductive outcomes, as determined by class, genotype, phenotype, environment and the population, which determines the course of evolution.

#### Definition 1

(*Population reproduction map*, $\mathbf {w}$) In addition to $x_i$ for the class, and $g_i$ for the genotype, of individual *i*, we will write the phenotype $a_i\in \mathcal {A}$ for one individual and for the whole population as $\mathbf {a}\in \tilde{\mathcal {A}}:=\bigcup _{k=1,2\ldots }\mathcal {A}^k$. We suppose that the reproduction of the population between each year is encapsulated by a single random variable $\mathbf {w}$, the *population reproduction map*, on the probability space $(\varOmega , \mathscr {F}, \mathbb {P})$ that takes an environment $e$ and class, genotype and phenotype arrays $\mathbf {x}$, $\mathbf {g}$, and $\mathbf {a}$ and returns an $|I|\times |X| \times |\mathcal {G}|$ array for the number of offspring-equivalents (per parental haploid set) of individual *i* in class $x'$ and with genotype $g'$. Our notational conventions already imply that a full notation for $w^i_{x',g'}$ would be

$$\begin{aligned} (\mathbf {w}(e,\mathbf {x},\mathbf {g},\mathbf {a}))^i_{x',g'} \;=\; w^i_{x',g'}(e,\mathbf {x},\mathbf {g},\mathbf {a}), \end{aligned}$$

which establishes the joint distribution of the $w^i_{x',g'}$ in terms of our single random function $\mathbf {w}$. Note that parental population arrays $\mathbf {x}$ and $\mathbf {g}$ appear as arguments of $\mathbf {w}$, but single classes and genotypes of offspring $x'$ and $g'$ appear as indices of the resulting array. For the avoidance of doubt, also note that $\mathbf {w}$ is a map from $(\varOmega , \mathscr {F}, \mathbb {P})$ to the space of maps from the possible values of $(e,\mathbf {x},\mathbf {g},\mathbf {a})$ to the possible rational arrays of size $|I|\times |X| \times |\mathcal {G}|$. These sets are complicated to notate, but the effect is that a given element of $\omega \in \varOmega$ specifies the precise reproductive consequences (including the outcomes of Mendelian segregation) of every possible environment and population structure. Thus, $\mathbf {w}$ is indeed a random variable on $(\varOmega , \mathscr {F}, \mathbb {P})$.

It is worth noting that the offspring population is characterised by $\sum _i{L_i \, \mathbf {w}^i}$ as an array over class and genotype spaces. When we need to treat this offspring population as the parents in the following year, we need to convert from this array description to the list of individuals that we use for parents. This conversion will be denoted by

$$\begin{aligned} (\mathbf {x}, \mathbf {g})' = {\text {extract}}\left( \sum _i{L_i \mathbf {w}^i }\right) . \end{aligned}$$

We will abuse this notation by using it to extract only $\mathbf {x}'$ where that is all we require. This conversion is needed because the attractions of the Price Equation include its treatment of the parent population as individuals, so we can make assumptions at that level; but the offspring population cannot be represented as individuals because we never make assumptions about how gametes combine to form individuals, which makes our results more general over genetic architectures. Thus, a somewhat awkward notational transition is a necessary consequence of the analytical strategy of the argument. The same conversion will be familiar to those readers who analyse categorical data, sometimes expressed with one individual per record, which contains the categorical variable values for that individual, and sometimes as tabulated data showing how many individuals share each given combination of variable values.

This extraction of individually indexed arrays from the tabulated array leaves the order of the individuals unspecified. We are about to make assumptions on $\mathbf {w}$ that make this indeterminacy entirely benign.

We make the following assumptions about $\mathbf {w}$. The effect of applying any permutation to the elements of each of the population arrays that comprise the second, third and fourth arguments of $\mathbf {w}$ is to apply the same permutation to the first dimension (which indexes the parental population) of the $|I| \times |X| \times |\mathcal {G}|$ array that $\mathbf {w}$ delivers. This assumption prevents the order in which the population is listed from carrying any information about geographical location or local environment, for example, apart from information carried purely by class, and ensures that just the set of triples $(x_i,g_i,a_i)_{i\in I}$ affects the outcome. It constrains how the environment can affect individuals, enforcing a symmetric treatment of them in distribution, and preventing the environment making special links between parents and offspring or between siblings. This assumption implies that the condition of pairwise exchangeability (assumed by Grafen 2002, 2006a, b; Batty et al. 2014, for fitness-maximixisation results) is met here too. It also prevents one individual affecting other individuals according to coancestral links. We further assume that the probability that the value of $\mathbf {w}$ implies a zero-size population in the next year is zero, as is the probability that the value of $\mathbf {w}$ renders a future zero-size population inevitable (such as an all-male population in a sexual species). This has much the same effect as conditioning our analysis on non-extinction, but is mathematically simpler. If we were interested in the chances of extinction, this assumption would be unhelpful: however, here our aim is to characterise the nature of adaptation in populations that do survive. A further technical assumption is that the entries of $\mathbf {w}$ are uniformly bounded, but this should be uncontroversial against a biological background.

A further, ‘genotype-through-phenotype’, assumption about $\mathbf {w}$ is that $\mathbf {g}$ does not affect $w^i_{x',+}(e,\mathbf {x},\mathbf {g},\mathbf {a})$, where the subscript ‘+’ indicates summing over the subscript $g'$. This asserts that genotypes affect the distribution of individuals’ offspring number and classes only via phenotypes; hence the inclusion of $\mathbf {a}$ in the list of arguments excludes any effect of $\mathbf {g}$ when the offspring are added up by class and parent, ignoring the offspring’s own genotypes. Of course, the genotypes do affect the *genotypes* of the offspring directly and not through phenotypes, and so while the sum is independent of $\mathbf {g}$ the summands are of course not. A formal expression of the assumption is

$$\begin{aligned} \sum _{g'}{w^i_{x',g'}(e,\mathbf {x},\mathbf {g}_1,\mathbf {a})} = \sum _{g'}{w^i_{x',g'}(e,\mathbf {x},\mathbf {g}_2,\mathbf {a})} . \end{aligned}$$

This assumption is regarded as simply requiring that the term ‘phenotype’ is being used in line with standard usage, and not as placing further restrictions. Without some formal requirement of this kind, a theory cannot make the genotype-phenotype distinction that is so central to our understanding of natural selection.

#### Remark 2

The function $\mathbf {w}$ lies at the heart of our model. It encapsulates how, given an environmental state *e*, a population of individuals with given phenotypes $a_i$, classes $x_i$, and genotypes $g_i$ will reproduce. In particular, for each individual it gives its share of individuals in each class and genotype in the next year. It is a random function, so it gives a probability distribution over all the possible outcomes. We have not placed many restrictions on $\mathbf {w}$, and so our conclusions are very general. If specialising to a particular case, it would be necessary to specify the functional form of $\mathbf {w}$, and also the functional form of the environmental process $\varPi$. Summarising, our first restriction is that the possibility of geographical, group or family structure is eliminated by the permutation assumption, except where that structure can be created by classes alone; our second prevents extinction from occurring; and our third insists that all effects of genotype, except on the actual genotypes of the offspring, must act through phenotype. Extending the framework to include kin interactions is the subject of current work, but the present framework does allow social interactions that depend on class, phenotype, and chance alone.

This very general definition of $\mathbf {w}$ is used to define two stochastic processes with $t=0, 1, 2, 3\ldots$, in the following sections.

### The neutral stochastic process

The neutral process tracks the class and genotype arrays $(\mathbf {x},\mathbf {g})^{(t)}$ of a population over time ($t=0, 1, 2\ldots$) in which there is a uniform ‘background’ phenotype, say $a_0$, in all years, so the genotypes are irrelevant to the evolution of the classes and the population size, though of course they remain relevant to the changes in genotypes. We assume that this process tends to an ergodic distribution (e.g. Rosenblatt 1971) over time that is independent of the starting conditions.

Let the random function $M:\bigcup _{k=1}^\infty {(\mathcal {G}\times X)^k} \rightarrow \tilde{\mathcal {G}}$ represent mutation: we assume it affects each individual’s genotype independently of all others and of all the other random variables in the model. The inclusion of *X* in the domain allows the mutation rate to be different for different classes, for example newborns versus surviving adults. The formal role of mutation is to maintain genetic variability in the neutral process, so that in the Taylor year there is genetic variability with which to create phenotypic variability. The choice of function *M* does not affect the reproductive values, as genotypes do not affect phenotypes in the neutral process, or classes. Thus, the effects of reproduction take $\mathbf {g}^{(t)}$ and produce ${\mathbf {g}^*}^{(t+1)}$, and mutation then takes place to complete the iteration as $\mathbf {g}^{(t+1)} = M((\mathbf {g}^*,\mathbf {x})^{(t+1)})$.

The neutral process has arbitrary initial conditions, which will be assumed not to affect the asymptotic distribution of the process, and we can summarise our informal discussion as

$$\begin{aligned} e^{(t+1)}&\sim \varPi (e^{(t)},\cdot ) \\ (\mathbf {x},\mathbf {g}^*)^{(t+1)}&={\text {extract}}\Big ( \sum _{i}{L_i \, \mathbf {w}^i(e^{(t)},\mathbf {x}^{(t)},\mathbf {g}^{(t)},a_0)} \Big ) \\ (\mathbf {x},\mathbf {g})^{(t+1)}&= (\mathbf {x}^{(t+1)},M((\mathbf {g}^*,\mathbf {x})^{(t+1)})). \end{aligned}$$

The assumption that there is an ergodic distribution of this process, independent of the initial conditions $(\mathbf {x}_0, \mathbf {g}_0)$, will allow us to define reproductive value. We denote the ergodic distribution by $\mathcal {D}(a_0)$, noting that our other assumptions are much too weak to prove existence.

It is convenient here to make a technical remark on the treatment of mutation. Unlike most developments of the Price Equation and the fundamental theorem, mutation is included in the model. This is required to maintain genetic variability asymptotically in a finite population, and so without it there would be no genetic variability to cause phenotypic variability, and neither kind of result would be of use. The introduction of mutation does introduce a complication, in that mutation will alter gene frequencies (for the Price Equation), and alter fitnesses (for the fundamental theorem). This is managed by placing mutation as the last step in each generation, and by applying results to the changes in gene frequencies before the mutation takes place. This elimination is contrasted with the approach of Tarnita and Taylor (2014) in the Discussion.

#### Definition 2

(*Expectation over neutral stochastic process,* $\mathbb {E}$) For use in following sections, we define the expectation $\mathbb {E}$ to be over the ergodic distribution $\mathcal {D}(a_0)$ of the neutral process. Conditioning on the initial state, this expectation is over chance events within one generation. Unconditionally, the expectation depends on the ergodic distribution and will be referred to as an expectation over the ‘ensemble’.

### The transition operator and reproductive value

Now, we implement the central idea that reproductive value should represent the fraction of an asymptotic gene pool that descends from an individual or a class—we will see in the discussion that this idea corresponds to Fisher (1930)’s verbal explanation but not to his mathematical formulation. This approach has been followed by Tuljapurkar (1989, 1990) and Rousset (2004) in models with continuing fluctuations in the population class distribution, and here we apply the same basic idea, in a finite population with class-based ploidies. New features here are permitting infinite classes, though we leave to future work exploring the consequences of permitting a continuous trait such as condition to be regarded as a class; and allowing the current population demographic structure to influence in an arbitrary way the per-capita reproduction of individuals in each class. We study this gene pool in the neutral stochastic process, in which all phenotypes equal $a_0$, and this allows us to focus, for this purpose, on the evolution of the class distribution only, ignoring the genetic aspect. Genotypes will have phenotypic consequences later in our second, ‘Taylor switch’, stochastic process.

For recursions in a phenotypically uniform population, we need to keep track only of the structure of the class distribution, which is captured by the population class array $\mathbf {x}$. This is because the phenotypes do not vary, and the ‘genotype-through-phenotype’ assumption then ensures that the genotypes do not affect the class distribution. Suppressing in its notation dependence on the value of the uniform phenotype, say $a_0$, we now define $\mathbf {x}'$, the population class array next year, as

6 $$\begin{aligned} \mathbf {x}' = \mathbf {x}'( e ,\mathbf {x}) \; = {\text {extract}}\Big (\sum _{i, g \in \mathcal {G}} {L_i \,\mathbf {w}^i(e,\mathbf {x}, \mathbf {g},a_0)}\Big ). \end{aligned}$$

It is important that this notation of $\mathbf {x}'$ as a function of the current environment and population class arrays will be used only under the assumption of phenotypic uniformity, though $\mathbf {x}'$ without arguments will continue to be used to denote the class array of the following year even when that assumption does not hold. Note that $(e,\mathbf {x})$ determines the distribution of $\mathbf {x}'$, and not its value, so $\mathbf {x}'(e,\mathbf {x})$ is still a random variable conditional on $(e,\mathbf {x})$.

In a model with stochastic environment and non-constant class distribution, the interpretation of reproductive value as fraction of an asymptotic gene pool can hold only if the reproductive values vary with the environment and with the class-distribution of the population, and these extra dependencies are key to our argument. Therefore we introduce the space $BM := BM(X\times E\times \tilde{X})$ of bounded, measurable, real-valued functions on $X\times E\times \tilde{X}$, where we recall that $\tilde{X}=\bigcup _{k=1,2\ldots } X^k$ is the space of non-zero, finite class arrays. An element of *BM* represents an allocation of a real number to each class, at each combination of environment and population class array. If reproductive value exists, it will be an element of *BM*.

It is convenient to define a function $n:X\times \tilde{X}\rightarrow \mathbb {N}\cup \{0\}$ that counts how many elements of an array $\mathbf {x}$ are equal to a particular element of *X*. Thus,

$$\begin{aligned} n(x,\mathbf {x}) := {\text {card}}\{j:x=x_j\} \end{aligned}$$

represents the number of individuals in the population in class *x*.

The reproductive values will be per-capita per-ploidy, and so we restrict ourselves to elements of *BM* that sum (over the population) to one, over its first argument, when weighted by each individual’s ploidy, for each pair of values of the second and third arguments, representing environment and population class array. To formalise, we define $\mathcal {Z}$.

#### Definition 3

($\mathcal {Z}$) Let the set of *candidate reproductive values*, $\mathcal {Z}$, be defined by

7 $$\begin{aligned} \mathcal {Z}:=&\Big \{f\in BM \;:\; f\ge 0, \; n(x,\mathbf {x})=0 \Longrightarrow f(x, e ,\mathbf {x}) = 0,\nonumber \\&\sum _i f(x_i, e ,\mathbf {x}) \cdot L_{x_i} = 1 \text { for all } e \in E \text { and } \mathbf {x}\in \tilde{X},\nonumber \\&f(x_i, e ,\mathbf {x}) = f(x_i, e ,\sigma (\mathbf {x})) \text { for all permutations}\, \sigma :\tilde{X}\rightarrow \tilde{X} \text { of the elements of}\, \mathbf {x}\Big \}. \end{aligned}$$

The population model inherent in $\mathbf {w}$ and $\varPi$ allows us to take a candidate function for reproductive value in the descendant year, and calculate what the parental year reproductive values would be, simply by adding up the expected value of the sum of a parent’s shares in the reproductive value of its offspring. The calculation is conditional on the information available in the parental generation. Thus, the candidate descendant reproductive value function defines a resulting parental reproductive value for each combination of class, environment, and population class array. We will therefore define a transition operator on $\mathcal {Z}$ that takes candidate descendant reproductive values as input and delivers parental reproductive values. If these are equal for any given candidate values, we will call these reproductive values. The first step in formalising is to define the class mean offspring arrays.

#### Definition 4

(*Class mean offspring array*, $\mathbf {u}^x$) It is useful to define for each parental class the mean array of offspring production retaining the division by offspring class but summing over genotypes, and we will denote this as $u^x_{y'}$, a random variable over $(\varOmega , \mathscr {F}, \mathbb {P})$ whose distribution is determined by $(e,\mathbf {x})$. We define it in a phenotypically uniform population with phenotype $a_0$. Then, in terms of our population reproduction map $\mathbf {w}$, we simply add up the output of the individuals in each class, and divide by their number, as follows

$$\begin{aligned} \mathbf {u}^x&= (u^x_{y'})_{y'\in X} = {\left\{ \begin{array}{ll} \frac{\sum _{i:x_i=x, g'}{w^i_{y',g'}( e ,\mathbf {x}, \mathbf {g}, a_0)}}{n(x,\mathbf {x})} &{} n(x,\mathbf {x})>0 \\ 0 &{} n(x,\mathbf {x})=0 \end{array}\right. }, \end{aligned}$$

where we note that the ordering of the elements of $\mathbf {x}$ does not affect the sum, by the permutation assumption on $\mathbf {w}$, and $\mathbf {g}$ does not affect the sum by the ‘genotype-through-phenotype’ assumption. $\mathbf {u}^x$ is the random array of mean offspring of members of class *x* in the next year, so $u^x_{y'}$ is the mean number of $y'$-offspring (evaluated as shares according to genetic contribution of haploid sets) of an *x*-parent, per parental ploidy. As with $\mathbf {x}'(e,\mathbf {x})$ this notation will be used only under the assumption of phenotypic uniformity.

We are now in a position to define the transition operator on $\mathcal {Z}$. For $f\in BM$ define the function $Tf:X\times E\times \tilde{X}\rightarrow \mathbb {R}$ by

8 $$\begin{aligned} (Tf)(x, e ,\mathbf {x}) :=&\int _E \mathbb {E}_{\mathbf {x}'} \bigg [\sum _{y'\in X} f(y', e ',\mathbf {x}'( e ,\mathbf {x})) \cdot L_{y'} \, u_{y'}^{x} \, \bigg ]\varPi ( e ,\mathrm {d} e ')\nonumber \\ =&\; \mathbb {E}_{ e '} \mathbb {E}_{\mathbf {x}'} \bigg [\sum _{y'\in X} f(y', e ',\mathbf {x}'( e ,\mathbf {x})) \cdot L_{y'} \, u_{y'}^{x} \,\bigg ]. \end{aligned}$$

Here $\mathbb {E}_{\mathbf {x}'}$ is expectation over the random variable $\mathbf {x}'$, with respect to the probability measure $\mathbb {P}$ on $\varOmega$ (the randomness in the reproduction of individuals in a given year, given the population structure) and $\mathbb {E}_{ e '}$ is with respect to the probability measure $\varPi ( e ,\cdot )$ on *E*, i.e., the conditional expectation of a function of the environment in year $t+1$, given that the environment at *t* is *e*.

The transition operator *T* takes a bounded function $f(x, e ,\mathbf {x})$ defined over the classes in each environment and population and maps it to a new function $(Tf)(x, e ,\mathbf {x})$ given by the expected value of the ploidy-weighted average of *f* over the offspring in year $t+1$ of parents who were in class *x*, in environment *e* and population $\mathbf {x}$, in year *t*. This use of *T* in essence extracts from the theory of Markov processes (see, e.g. Rosenblatt 1971) just what we need for current purposes.

#### Remark 3

Since *f* takes a value at each point of $X\times E\times \tilde{X}$ and *f*, *u* and $\varPi (e,\cdot )$ are bounded, *T* is well defined as a bounded linear operator on *BM*.

It is important for our argument that a candidate function, which sums to one over the population, is transformed by *T* into a parental function that also sums to one. This corresponds to the idea that reproductive value sums to one over each population state, and makes it possible to prove under various kinds of additional assumptions that there is a fixed point of *T* in *Z*. The following lemma is proved in Appendix A.

#### Lemma 1

The set $\mathcal {Z}$ is non-empty and *T*-invariant, that is, $(Tf) \in \mathcal {Z}$ for all $f\in \mathcal {Z}$.

#### Definition 5

(*Reproductive value*, $\phi ^*, \phi$) Suppose there exists a fixed point of *T* in $\mathcal {Z}$, that is, a function $\phi ^*\in \mathcal {Z}$ such that $(T\phi ^*) = \phi ^*$. Given such a function $\phi ^*$, we define the *reproductive value per haploid set* of an individual in class $x\in X$, living in an environment $e \in E$ amidst a population described by the array $\mathbf {x}\in \tilde{X}$ to be $\phi ^*(x, e ,\mathbf {x})$. We now define the corresponding *reproductive value* of the individual (that is, *not* per haploid set) as

$$\begin{aligned} \phi (x, e ,\mathbf {x}) \;:=\; L_x\,\phi ^*(x,e,\mathbf {x}). \end{aligned}$$

Where it is clear from the context we will sometimes write $\phi _x$ for $\phi (x, e ,\mathbf {x})$. Reproductive value relies on the existence of an asymptotic distribution of the process, and measures for an individual (or, by summing, for a class) in a given year the fraction of the gene pool in a very distant generation that derives from that individual (or class) in that year.

It may be the case that no such fixed point $\phi ^*$ exists or that $\phi ^*$ is non-unique: the present level of abstraction is simply too high to prove existence or uniqueness in general. The biological interpretation is discussed elsewhere (Grafen 2006b). Despite this apparent weakness, defining reproductive value in this way is a powerful tool and in simple models the construction of reproductive value is often straightforward (Grafen 2006b). For a case where existence can be proven see Appendix B. This definition differs from previous mathematical definitions of reproductive value, but we argue in the discussion that here we have implemented Fisher’s verbal, asymptotic characterisation, which is appropriate for a long-run average growth rate of zero, but allowing for year-to-year variation. Fisher (1930) also had a mathematical, demographic definition, according to which the total reproductive value of the population increased (or decreased) at a constant exponential rate over time, as do others such as Engen et al. (2009). For models with a constant population size (e.g. Grafen 2006b; Barton and Etheridge 2011; Batty et al. 2014), the distinction between demographic and asymptotic gene pool definitions is without a significant difference. There are definitions that maintain the sum of reproductive value over the population as one, and include fluctuating demography. Compared to Tuljapurkar (1989), our model has an infinite class set *X*, and allows the relative successes of individuals in different classes to depend on the class distribution (as opposed to assuming, in his notation, that $X_{t+1}$ is independent of $Y_t$). Compared to Rousset (2004, eqn 10.15), our model does not assume there is a typical individual whose fitness depends only on its own trait value, the mean in its group, and the mean in the population. In particular our formulation allows individuals in different classes to have quite different fitness functions, in contrast to Rousset’s main interpretation of his population subdivisions as demes. Rousset and Ronce (2004, eqn 8) define reproductive values for demes in a structured population with fluctutating demography, and allow arbitrary dependence of individual offspring numbers on the demographic state. Their definition is thus very similar to ours, apart from the possibiity of infinite classes, and some minor details. These technicalities make a difference, but much more significant is Rousset and Ronce’s use of reproductive values: ‘The only point of the choice of [reproductive values] as weights is that the expected change in allele frequency is null in the neutral model [...], which simplifies later computations’ (Rousset and Ronce 2004, p. 131). Here, we use reproductive values in a more general model of how individual fitnesses are determined, and we define fitness for individual organisms in such as way as to establish a Price Equation and a fundamental theorem of natural selection: thus we choose a route foregrounding individual organisms rather than gene frequencies. As mentioned in the Introduction, this choice comes at the significant cost of losing dynamic sufficiency.

#### Remark 4

Some key properties of reproductive value are as follows:

(i) The reproductive value of an individual in class *x* in a given year is equal to the expected value of the sum of a class *x* individual’s genetic share (i.e., $\frac{1}{2}$ for diploids) of the reproductive value of its offspring in the following year, plus the reproductive value due to its surviving self. It is important that the class reproductive values in the parental population will generically be different from those in the descendant population (i.e. ($\phi (x, e ,\mathbf {x}))_{x\in X} \ne (\phi (x, e ',\mathbf {x}'( e ,\mathbf {x}))_{x\in X}$), because the population class array will have changed ($\mathbf {x}\ne \mathbf {x}'( e ,\mathbf {x})$, even allowing permutations) and the environment will be different ($e\ne e'$).
(ii) Reproductive values sum to one over the population, whatever values are taken by the population class array and the environment, and so they can be used as weights to construct convenient population averages.

In the next section, a martingale property of reproductive value is established.

### Reproductive value as a martingale

This section proves a theorem that links reproductive value in the neutral stochastic process to martingale theory. To deal with asymptotic gene pools, we extend our notation slightly by adding a year index, so that $(u^{x,(t)}_{y'})_{y'\in X}$ is the class mean offspring array for class *x* in year *t*. Let $H^{(t)}$ be the transition matrix derived from $\mathbf {w}( e ^{(t-1)},\mathbf {x}^{(t-1)},\mathbf {g}^{(t-1)},a_0)$ that converts the class-distribution of the population from the beginning of year $t-1$ to the beginning of year *t*. $H^{(0)}$ is understood to be the initial population arrays $(\mathbf {x}^{(0)})$, so that the sequence $(e^{(k)}, H^{(k)})_{k=0,1\ldots t}$ effectively defines the whole history of the process (population and environment) up to the start of year *t*. We may ignore the genetic arrays $\mathbf {g}$ because the genetics has no influence on the course of the neutral stochastic process, and in particular on the class arrays.

A martingale is a sequence of random variables measurable with respect to a filtration, which is a sequence of nested sigma-algebras, which grow more refined as the years go by, representing increasing knowledge as random variables are realised. In this case, we define $(\mathcal {H}^{(t)})_{t=0,1,2\ldots }$ as the filtration generated by $(e^{(t)},H^{(t)})_{t=0,1,2\ldots }$. Conditioning on $\mathcal {H}^{(t)}$ is equivalent to conditioning on all values of those generating variables, up to and including year *t*, and they have been chosen to make this the entire history of the process. This significantly includes $u^{x,(t)}_{y'}$, which allows us to keep track of which ancestral classes have contributed to each descendant class in each year.

The quantity at the centre of the theorem is an individual’s share of reproductive value in succeeding years. An individual in class *x* will have descendants in a given year, and it has a share of each descendant according to the ancestral links. Those fractions of the reproductive values of the descendants can be added up and regarded as the reproductive value descending from the individual. This depends on the realisation of random variables representing how many offspring each class of individual has. To improve readability, we drop for this section the convention of adding a prime to offspring subscripts. We make formal definitons as follows.

#### Definition 6

($c^{k,l}_{x,z}$) Let $c^{k,l}_{x,z}$, for $k,l=0,1,2\ldots$ and $x,z\in X$ be recursively defined by

$$\begin{aligned} c^{k,k}_{x,z}&= {\left\{ \begin{array}{ll} 1 &{} x=z \\ 0 &{} x\ne z \end{array}\right. } \\ c^{k,k+l+1}_{x,z}&= \sum _y{\, L_y \, u^{y,(k+l)}_z \, c^{k,k+l}_{x,y}}, \end{aligned}$$

and note that $c^{k,k+l}_{x,z}$ is a random variable over $\mathcal {H}^{(k+l)}$. This quantity represents the expected number-equivalent of descendants in class *z* in year $k+l$ of one individual in class *x* in year *k*. The reproductive value in year *t* descended from an individual in class *x* in year 0 may now be defined by

9 $$\begin{aligned} R^{(t)}_x := \sum _{z\in X}{\,c^{0,t}_{x,z}\, L_z \, \phi ^*(z, e ^{(t)},\mathbf {x}^{(t)})}. \end{aligned}$$

This does not represent the actual fraction of the gene pool in year *t* descending from the individual, because that will depend on the details of segregation in the intervening years. In conventional diploidy, the fraction of a grandoffspring’s genome deriving from a grandparent will not be exactly a quarter, but will vary depending on the outcomes of meiosis, and will be different at different loci. However, in expectation over fair Mendelian segregation, the expected fraction is a quarter at each autosomal locus. Similarly, $R^{(t)}_x$ is a random variable over $\mathcal {H}^{(t)}$ that tracks all the other sources of randomness, but takes expectations over Mendelian segregation.

These definitions allow us to state

#### Theorem 1

(Reproductive value as a martingale) In the neutral stochastic process, the random process $(R^{(t)}_x)_{t=0,1,2\ldots }$ is a *martingale* with respect to the filtration $(\mathcal {H}^{(t)})_{t=0,1,2\ldots }$, for each $x\in X$.

#### Proof

To establish the martingale property it will suffice to prove

10 $$\begin{aligned} \mathbb {E}_{ e ^{(t+1)}} \mathbb {E}_{\mathbf {x}^{(t+1)}} \big [\;R^{(t+l+1)}_x - R^{(t+l)}_x\; \big | \; \mathcal {H}^{(t)} \;\big ] = 0 \end{aligned}$$

for all $$t,l\ge 0$$.

As a preliminary, we rewrite the formula (8) defining *T* applied to the fixed point $\phi ^*$, to make explicit the conditioning on the state of the process and employ time superscripts. Formally,

11 $$\begin{aligned} \phi ^*(x, e ^{(k)},\mathbf {x}^{(k)}) =&\; (T\phi ^*)(x, e ^{(k)},\mathbf {x}^{(k)}) \nonumber \\ =&\; \mathbb {E}_{ e ^{(k+1)}} \mathbb {E}_{\mathbf {x}^{(k+1)}} \bigg [\sum _{y\in X} \phi ^*(y, e ^{(k+1)},\mathbf {x}^{(k+1)}) \cdot L_y \, u^{x,(k)}_y \; \bigg | \, \mathcal {H}^{(k)}\, \bigg ]. \end{aligned}$$

Now expand $R^{(t+l+1)}_x$, using the definition of *c*, to find an equality between random variables on $\mathcal {H}^{(t+l+1)}$ as follows

$$\begin{aligned} R^{(t+l+1)}_x&= \sum _{z\in X}{\,c^{0,t+l+1}_{x,z}\, L_z \, \phi ^*(z, e ^{(t+l+1)},\mathbf {x}^{(t+l+1)})} \\&= \sum _{z\in X}{\,\sum _{y\in X}{\, L_y \, u^{y,(t+l)}_z \, c^{0,t+l}_{x,y}} \, L_z \, \phi ^*(z, e ^{(t+l+1)},\mathbf {x}^{(t+l+1)})} \\&= \sum _{y\in X}{\,L_y \, c^{0,t+l}_{x,y}}\,\sum _{z\in X}{\, \, u^{y,(t+l)}_z \, \, L_z \, \phi ^*(z, e ^{(t+l+1)},\mathbf {x}^{(t+l+1)})}. \end{aligned}$$

We take expectations of both sides with respect to $\mathcal {H}^{(t+l)}$. The inner sum is the only remaining random variable on the RHS, and the expected value of that inner sum equals $\phi ^*(y, e ^{(t+l)},\mathbf {x}^{(t+l)})$ by Eq. (11). The Definition (9) of $R^{(t)}_x$ then allows us to obtain

$$\begin{aligned} \mathbb {E}_{ e ^{(t+l+1)}} \mathbb {E}_{\mathbf {x}^{(t+l+1)}} \big [ \, R^{(t+l+1)}_x \, \big |\, \mathcal {H}^{(t+l)} \, \big ] = \sum _{y\in X}{\,L_y \, c^{0,t+l}_{x,y}\,\phi ^*(y, e ^{(t+l)},\mathbf {x}^{(t+l)})} = R^{(t+l)}_x. \end{aligned}$$

It now remains to observe that we can take expectations with respect to $\mathcal {H}^{(t)}$ of both sides, and that the inner expectation on the LHS collapses into the new expectation by the tower property (see, e.g. Schechter 1997), leading to

$$\begin{aligned} \mathbb {E}_{ e ^{(t+1)}} \mathbb {E}_{\mathbf {x}^{(t+1)}} \big [ \, R^{(t+l+1)}_x \, \big | \,\mathcal {H}^{(t)} \, \big ] = \mathbb {E}_{ e ^{(t+1)}} \mathbb {E}_{\mathbf {x}^{(t+1)}} \big [ \, R^{(t+l)}_x \, \big | \, \mathcal {H}^{(t)} \, \big ], \end{aligned}$$

which immediately implies Eq. (10), completing the proof. $\square$

#### Remark 5

The theorem shows that the fraction of the total reproductive value of the population in year *t* tracing back to an individual in year 0 is a martingale, i.e. conditional on the realised value of that reproductive value in year *t*, the expected value at every future year is the same as its value in year *t*. There is no novel biological point here. Rather, it is a formal justification for our informal assertion that, under Mendelian mixing, the reproductive value of Definition 5 does represent the expected fraction of the gene pool of some distant future generation that traces its descent to an individual or set of individuals in some earlier year.

### The Taylor switch stochastic process

The role of the Taylor switch process is discussed at the start of Sect. 2, and the process itself $(\mathbf {x},\mathbf {g})^{(t)}_{t=0,1,2\ldots }$ is the same as the neutral process except for two properties. The state at $t=0$ is random, being drawn from the ergodic distribution of the neutral process. The second difference is that in year $t=0$, the phenotypes are determined by an arbitrary (measurable) function of the genotypes and classes, contrasting with all other years in the Taylor switch process and with all years in the neutral process, in which the phenotype is uniformly the background phenotype $a_0$. This allows us to study the effect of an arbitrary genotype and class to phenotype map, on the basis of a uniform background phenotype in all other years. One important property is that the arbitrary genotype-phenotype map is tested in a random year in the ensemble distribution created by the uniform phenotype. Another is that the map creates differences in frequencies of genes and classes which are then played out in, and can be evaluated according to the reproductives values calculated from, a population with the background phenotype.

We recall the definition of $\mathcal {D}(a_0)$ in Sect. 2.2, and then we formally define the process by

$$\begin{aligned} ( e , \mathbf {x},\mathbf {g})^{(0)}&\sim \mathcal {D}(a_0) \\ a^{(t)}_i&= {\left\{ \begin{array}{ll} h(x^{(0)}_i, g^{(0)}_i) &{} t=0 \\ a_0 &{} t\ge 1 \end{array}\right. } \\ e^{(t+1)}&\sim \varPi (e^{(t)},\cdot ) \\ (\mathbf {x},\mathbf {g}^*)^{(t+1)}&= {\text {extract}}\Big ( \sum _{i}{L_i \, \mathbf {w}^i(e^{(t)},\mathbf {x}^{(t)},\mathbf {g}^{(t)},\mathbf {a}^{(t)})} \Big ) \\ (\mathbf {x},\mathbf {g})^{(t+1)}&= (\mathbf {x}^{(t+1)},M((\mathbf {g}^*,\mathbf {x})^{(t+1)})). \end{aligned}$$

The lack of subscript on *h* implies that each individual’s phenotype depends only on its own genotype and class and not on the genotypes or classes of other individuals. The formalism allows an arbitrary function *h*, and the Price Equation and the fundamental theorem to be proved below are focussed on the consequences for natural selection of the function *h* that links genotypes to phenotypes.

#### Definition 7

(*Extension for* $\mathbb {E}$ *to Taylor year*, $\mathbb {V}$) For use in the following section, we extend the definition of the expectation $\mathbb {E}$, so that as well as taking expectations over the ergodic distribution $\mathcal {D}(a_0)$ of the neutral process, it also takes expectations over the phenotypes created by the classes, genotypes and environment at $t=0$ of the Taylor switch process. This allows genetic effects on phenotype and fitness, so selection can occur. The corresponding variance $\mathbb {V}$ is also defined: note it will be used only in Theorems 3 and 4.

## The Price equation

As a preliminary, we introduce some useful notation. One Price Equation for the Taylor year is then proved that takes the initial state as known, and then a further Price Equation is proved taking expectations over the initial state. Each version is used in Sect. 4 to prove a corresponding version of Fisher’s fundamental theorem of natural selection.

### Population means and covariances

We introduce Price’s notation (Price 1972), as slightly extended (Grafen 2015b), for weighted population means and covariances, which will allow us to express results briefly and clearly. The subscript *i* that runs over members of the population is suppressed. All the weighting is with respect to $\phi$, so we are weighting by the individual’s reproductive value. This corresponds to the weighting in Grafen (2015b), as his weights correspond to our $L_i \phi ^*_i$.

Given functions $f,g,h:I\rightarrow \mathbb {R}$, we define ${\text {ave}}$, ${\text {cov}}$ and ${\text {var}}$, as

$$\begin{aligned} {\text {ave}}_{h} f&= \frac{\sum _i{h_i f_i}}{\sum _i{h_i}} \\ {\text {cov}}_{h}\left( f,g\right)&= {\text {ave}}_{h} (fg) - ({\text {ave}}_{h} f )( {\text {ave}}_{h} g ) \\ {\text {var}}_{h} f&={\text {cov}}_{h}\left( f,f\right) . \end{aligned}$$

A further definition of $\varDelta ^*$ involves the notation of $'$ to mean the same corresponding value in the next year, as follows,

$$\begin{aligned} \varDelta ^* {\text {ave}}_{h} f = {\text {ave}}_{h'} {f^*}' - {\text {ave}}_{h} f , \end{aligned}$$

which indicates that both the weights and the main variables take a prime. The superscript $^*$ indicates before mutation, as in the definition of the stochastic processes, implying that while the gene frequency in the current year will be based on the genotype array $\mathbf {g}$, the gene frequency in the next year is based on the pre-mutation genotype array ${\mathbf {g}^*}'$.

There are two reasons to use this notation, rather than to use standard expectation notation by making these simple expectations conditional on the full demographic state of the population and on the environment. First, these population, or statistical, means and covariances are very different from the probabilistic versions used elsewhere in this paper, a distinction drawn elsewhere (van Veelen 2005). Employing the standard notation to represent a population mean implies an ancillary and somewhat artificial randomness of selecting one individual from the population at random, and furthermore that selection should be done weighting by reproductive value; whereas the other randomness, such as caused by weather, is more naturally understood as random, at least by biologists. The terms ‘population mean’ and ‘population covariance’ will be used when it is helpful to make verbally that mathematical distinction. Second, biologists who think about real populations will find it useful to understand that ${\text {ave}}$, ${\text {cov}}$ and ${\text {var}}$ represent quantities that might be measured in a population, while the other expectations involve averaging over environmental uncertainty. This formulation allows all population means and population covariances to have the same weights, whereas an earlier, less satisfactory treatment by Batty et al. (2014) used different weights over the same population (see discussion in Grafen 2015b). Expectations over random processes have already been defined using $\mathbb {E}$, so keeping the two kinds of means notationally distinct. The simple and easily interpretable form of the final results is also a consequence of the use of this notation.

### The Price equation

We proceed to our next result, a Price (1970)—type covariance equation for the change of gene frequencies over the Taylor year, before mutation occurs. The general modelling philosophy, including more on the Price Equation approach, is outlined in the ‘Reproductive value’ section of the Discussion. So far, the array of genotypes $\mathbf {g}$ has been carried along formally in the definition of the two stochastic processes, and now we begin to use it. Suppose we have a set $\mathcal {K}$ of alleles, associated with the set $\tilde{\mathcal {G}}$ of genotype arrays. A genotype array has a typical element $g_i$, which is a simple element considered as a genotype. However, a genotype may itself be considered as an array of allele frequencies, and we therefore define $g_i^k$ to be the fraction of alleles in individual *i* at the locus containing allele $k\in \mathcal {K}$ which are in fact allele *k*. The alleles may be at an arbitrary number of loci, and have an arbitrary linkage map, and arbitrary linkage disequilibrium. We assume, however, that all the loci belong to the same coreplicon (Cosmides and Tooby 1981—see discussion in Sect. 2.1). These are standard assumptions for the Price Equation and the fundamental theorem (see, e.g. Price 1970; Ewens 2004). We also assume that meiosis is fair and transmission is unbiased. We take this to mean that the expected frequency of any allele, and so the expectation of any linear combination of allele frequencies, in a gamete is equal to its value in the individual producing the gamete: under this condition, the second, population average, term in the usual Price Equation (Price 1970) will be exactly zero in expectation.

We apply the Price Equation to the transition from $t=0$ to $t=1$ in the Taylor switch stochastic process, that is, the Taylor year, as that is the only moment at which genotype affects phenotype. To exclude the effect of mutation during the year, we compare $\mathbf {g}$ to the pre-mutation genotype array ${\mathbf {g}^*}'$. We prove a result that conditions on the values of $( e ,\mathbf {x},\mathbf {g})^{(0)}$, and then a second that takes an expectation over the initial conditions of the stochastic process, that is, over the ergodic distribution of the neutral stochastic process. We will drop the year superscript where possible, and continue to follow the notation of a prime for ‘next year’, such as $\mathbf {x}'$.

#### Definition 8

(*p*-*score*, $\pi$) We say that a function $p:I\rightarrow \mathbb {R}$ is a *p-score* if *p* is a linear combination of allele frequencies. In order to weight a *p*-score *p* by reproductive value, we define $\pi :X\rightarrow \mathbb {R}$ by setting $\pi _x$ to be the sample mean of *p* over members of class *x*,

$$\begin{aligned} \pi _x := \left\{ \begin{array}{cl} \frac{\sum _{i\,:\, x_i=x} p_i}{n(x,\mathbf {x})} &{} \qquad \text {if }n(x,\mathbf {x}) \ne 0,\\ 0 &{}\qquad \text { otherwise}. \end{array}\right. \end{aligned}$$

#### Remark 6

As in previous work on reproductive value in structured populations, the important quantity for the evolution of the population is not the raw average of gene frequencies or *p*-scores but the reproductive-value-weighted mean of these quantities. From the perspective of a gene, its presence in individuals with greater reproductive value (those more likely to pass it on in greater numbers) is proportionally more important to the spread of that gene than its presence in low-value individuals and so the correct metric of the gene’s spread is to weight every individual by their reproductive value. This was stated as a principle by Fisher (1930, p. 36) in his exposition of the fundamental theorem and reinforced for stochastic demography by Rousset and Ronce (2004). By weighting in this way, we capture the effect of short term frequency change on expected long term trends.

Given a *p*-score *p*, the *reproductive-value-weighted mean p-score* of the population with class array $\mathbf {x}$ in an environment $e \in E$ is

12 $$\begin{aligned} {\text {ave}}_{\phi } p \; = \; \sum _{x\in X} \pi _x \, n(x,\mathbf {x}) \, \phi (x, e ,\mathbf {x}) \; = \; \sum _{i} \,p_i \, \phi (x_i, e ,\mathbf {x}) . \end{aligned}$$

We also need an expression for the weighted mean *p*-score in the population next year before mutation. In expectation over fair meiosis, the gene frequencies of gametes are equal to the gene frequency of their parent. For each class $y'\in X$ the expected sum of *p*-scores of offspring in a class $y'$ is equal to the sum over the population of the parents’ *p*-scores multiplied by their number of offspring in class *y*,

$$\begin{aligned} \sum _{i\in I}\, p_i \, L_i \, w_{{y',+}}^{i}. \end{aligned}$$

Whence the weighted mean *p*-score in the next year’s population before mutation, whose class and genotype arrays we will denote $\mathbf {x}'$ and ${\mathbf {g}^*}'$ as a convenient shorthand for $\mathbf {x}^{(1)}$ and $\mathbf {g}^{*(1)}$, is a random variable over the natural product of $(\varOmega , \mathscr {F}, \mathbb {P})$ and *E*, whose expectation satisfies

13 $$\begin{aligned} \mathbb {E}\big [ {\text {ave}}_{\phi '} {p^*}' \big ] \; = \; \mathbb {E}\bigg [\sum _{y'\in X} \phi (y', e ',\mathbf {x}') \cdot \bigg (\sum _{i\in I} \,p_i \, L_i \, w_{{y',+}}^{i}\bigg ) \bigg ], \end{aligned}$$

where $$ e '$$ is the next year’s environment, an *E*-valued random variable with distribution $$\varPi (e, \cdot )$$. A formula for $${\text {ave}}_{\phi '} {p^*}' $$ without a surrounding expectation would contain an additional right hand term for biassed transmission and Mendelian uncertainty, but this term equals zero in expectation on our assumption of unbiassed transmission, and so does not appear in Eq. (13).

#### Definition 9

(*Fitness*, $\mathcal {F}_i$) For an individual $i\in I$, we define its *fitness* to be the sum of (*i*’s share of) the reproductive values of the offspring of *i*, minus the reproductive value *i* itself expects, and this difference is expressed relative to the reproductive value *i* itself expects. (An informal version of this definition is given in Eq. (1) in the introduction.) Thus,

$$\begin{aligned} \mathcal {F}_{i} \; := \frac{ L_i \; \sum _{y'\in X}{ \phi (y', e ',\mathbf {x}') \, w_{{y',+}}^{i}} \; - \; \phi (x_i, e ,\mathbf {x})}{\phi (x_i, e ,\mathbf {x})}, \end{aligned}$$

where $e '\in E$ is again the next year’s environment. Parental ploidy appears in each of the three terms, twice implicitly as $\phi _i = L_i \phi ^*_i$, and so cancels out, showing fitness has no tendency to increase with ploidy. Thus, we arrive at a view of fitness as an individual measure of the tendency to increase the representation of each allele an individual possesses, relative to the expectation for its class.

#### Remark 7

Consider what we know about individual *i*’s contribution to the asymptotic gene pool before and after the actions in the Taylor year are performed. Beforehand, we have only the expected share according to the individual’s class, namely $\phi (x_i, e ,\mathbf {x})$, while afterwards we know the individual’s shares in descendants and their classes, and we know the environment next year, so our best guess at the contribution is $L_i \sum _{y'\in X}{ \phi (y', e ',\mathbf {x}') \, w_{y'}^{i}}$. The definition of fitness we shall adopt (following Fisher 1930, as articulated by Grafen 2015a), is ‘(after–before)/before’, that is, the proportional change in our expectation of *i*’s contribution to the asymptotic gene pool as a result of moving forward one year in our state of knowledge. By this definition, fitness is a random variable of both the uncertainty in the individual’s reproductive outcome $(\varOmega ,\mathscr {F},\mathbb {P})$ and in the next year’s environment $(E,\varSigma ,\varPi (e,\cdot ))$. Though intimately related, it is distinct from reproductive value. For the Taylor year only, we allow an arbitrary genotype-phenotype map, and so *fitness* is a function of the individual not the class. During that year, individuals may differ from their class-mates, including for genetic reasons, which creates the possibility of natural selection. We record two technical statements about $\mathcal {F}$ in a lemma.

#### Lemma 2

(Technical properties of fitness) In general over the Taylor year,

$$\begin{aligned} {\text {ave}}_{\phi } \mathcal {F} =0. \end{aligned}$$

Further, if the phenotype in the Taylor year is uniformly the background phenotype, which will certainly be implied when $h(x,g)=a_0$ for all *x*, *g*, then

$$\begin{aligned} \mathbb {E}\big [\, \mathcal {F}_i \, | e ,\mathbf {x},\mathbf {g}\big ] = 0\quad \forall i. \end{aligned}$$

#### Proof

The first statement holds because

$$\begin{aligned} {\text {ave}}_{\phi } \mathcal {F} = \sum _i{\phi _i \mathcal {F}_i} \; = \; \sum _i{ L_i \; \sum _{y'\in X}{ \phi (y', e ',\mathbf {x}') \, w_{{y',+}}^{i}}} \; - \; \sum _i{\;\phi (x_i, e ,\mathbf {x})} \end{aligned}$$

and we proceed to show that both sums equal 1, each on the grounds that the total reproductive value in a population always equals 1, which is also why no denominator for ‘sum of weights’ is required. The second sum is the total reproductive value of individuals in the parental year. The first sum is the sum in the offspring year, because $L_i w_{{y',+}}^{i}$ equals the shares of offspring in class $y'$ of individual *i*, so $L_i w_{{y',+}}^{i} \phi (y', e ',\mathbf {x}')$ equals the corresponding shares in the reproductive values. When these shares are summed over all offspring classes $y'$ and over all parents *i*, they amount to the total reproductive value in the offspring year, establishing the statement.

The second statement holds because in a phenotypically uniform population, Definition 4 makes $u_{y'}^{x_i}$ the average of the $w_{y',+}^i$ for individuals *i* in class *x*. The permutation assumption on $\mathbf {w}$ now ensures that $\mathbb {E}[w_{y',+}^i | e ,\mathbf {x},\mathbf {g}]=u_{y'}^{x_i}$. The fixed-point property for reproductive value in Definition 5 then implies

$$\begin{aligned} L_i \; \sum _{y'\in X}{ \phi (y', e ',\mathbf {x}') \, w_{{y',+}}^{i}} \; = \; \phi (x_i, e ,\mathbf {x}), \end{aligned}$$

which shows that the expectation of the numerator of the definition of fitness in Definition 9 equals zero. $\square$

#### Remark 8

(Possibly infinite fitnesses) It is possible that $\phi _i=0$ in the Definition 9 of fitness, when the numerator is positive. This arises when a class that, under the uniform phenotype, obtains no reproductive value through offspring or survival, manages under the Taylor switch to obtain some reproductive value. Fitness for that class is then in a sense ‘infinite’ and so technically undefined, but infinite fitness accurately represents the ratio of advantage compared to the background phenotype. The theorems still hold in a biologically meaningful way, as the gene frequencies change in a finite way, representing a balance between the zero reproductive value of a class (based on its failure to reproduce with the background phenotype) and its infinite fitness.

We are now in a position to prove

#### Theorem 2

(The Price equation) The expected change in the reproductive-value-weighted mean of an arbitrary *p*-score *p*, over the Taylor year, conditioning on the state of the population at $t=0$, and before mutation, is

14 $$\begin{aligned} \mathbb {E}\big [ \,\varDelta ^* {\text {ave}}_{\phi } p \,\big ] \;=\; {\text {cov}}_{\phi }\left( \,p, \, \mathbb {E}[\,\mathcal {F}\,]\, \right) . \end{aligned}$$

#### Remark 9

The covariance expression in Eq. (14) exhibits the Price equation in parallel form to the original (Price 1970, 1972), with three important differences. First, by assuming unbiassed transmission, we have no second term on the right hand side representing deviations: this result is exact because we are taking expectations over Mendelian segregation. This difference would be a disadvantage if we aimed to study meiotic drive, for example, but we are restricting the loci to belong to a single coreplicon (Cosmides and Tooby 1981). Second, this form holds for a class-structured population by adopting class weights that satisfy a principle of ‘neutrality’, namely that whenever all the current genetic variability is currently phenotypically neutral, the additive genetic variance in fitness equals zero. Neutrality in a similar sense has been recognised as a property of reproductive value since Taylor (1990), and as the main property under stochastic demography by Rousset (2004) and Rousset and Ronce (2004). Grafen (2015b) shows in a deterministic model that guaranteeing neutrality requires using reproductive values as weights. The third, novel, aspect here is that the result holds under great generality including stochastic environment and continuing fluctuations in class distribution, with *exactly the same form* except that the change on the left hand side is an expectation, as is the fitness inside the covariance operator on the right hand side. It is remarkable that such a simple equation holds under stochastic demography, and greatly extends the part that the combination of the Price equation and Fisher’s reproductive value can play in our understanding of natural selection.

#### Proof

First note that the sum of $p_i - \pi _{x_i}$ is zero over any class, and therefore, since $i\mapsto \mathbf {u}^{x_i}$ and $L_i$ are constant on classes we have

$$\begin{aligned} \sum _{i} \,\big (\pi _{x_i} - p_i \big ) \, L_i \, \mathbf {u}^{x_i} \;=\; 0. \end{aligned}$$

Briefly employing the notation $\mathbf {w}_+^i$ for the array of the classes of the offspring of individual *i* (that is, for $(\sum _{g'}{w^i_{x',g'}})_{x'\in X}$), we can now write the array $\sum _{i} p_i \, L_i \, \mathbf {w}_+^{i}$ from the expression for the average *p* score among descendants (13) in the following form

15 $$\begin{aligned} \sum _{i}\, p_i \, L_i \, \mathbf {w}_+^{i} \;&=\; \sum _{i}\, p_i \, L_i \, \mathbf {w}_+^{i} \;+\; \sum _{i} \,\big (\pi _{x_i} - p_i \big ) \, L_i \, \mathbf {u}^{x_i} \nonumber \\&=\; \sum _{i}\, p_i \, L_i \, \big (\mathbf {w}_+^{i} - \mathbf {u}^{x_i}\big ) \;+\; \sum _{x\in X} \,\pi _{x} \,n(x,\mathbf {x}) \, L_x \, \mathbf {u}^{x}. \end{aligned}$$

Substituting (15) into (13) and swapping the order of summation gives

16 $$\begin{aligned} \mathbb {E}\big [ {\text {ave}}_{\phi '} {p^*}' \big ] \;&=\; \int _E \mathbb {E}_{\mathbf {x}'}\Bigg [\sum _{y'\in X} \phi (y', e ',\mathbf {x}') \cdot \bigg (\sum _{i} p_i \, L_i \, w_{y',+}^{i}\bigg ) \Bigg ]\,\varPi ( e , \mathrm {d} e ')\nonumber \\&=\; \int _E \mathbb {E}_{\mathbf {x}'}\Bigg [\sum _{y'\in X} \phi (y', e ',\mathbf {x}') \cdot \bigg (\sum _{i} p_i \, L_i \, \big (w_{y',+}^{i} - u^{x_i}_{y'} \big ) \bigg ) \nonumber \\&\quad +\; \sum _{y'\in X} \phi (y', e ',\mathbf {x}') \cdot \bigg (\sum _{x\in X} \pi _x \,n(x,\mathbf {x}) \, L_x \, u_{y'}^{x} \,\bigg ) \Bigg ]\,\varPi ( e , \mathrm {d} e ')\nonumber \\&=\; \sum _{i} \, p_{i} \, L_{i} \cdot \int _E\mathbb {E}_{\mathbf {x}'}\bigg [\sum _{y'\in X} \phi (y', e ',\mathbf {x}') \cdot \big (w_{y',+}^{i} - u^{x_i}_{y'} \big ) \bigg ]\,\varPi ( e , \mathrm {d} e ') \nonumber \\&\quad +\; \sum _{x\in X} \pi _x \, n(x,\mathbf {x}) \, L_x \cdot \int _E \mathbb {E}_{\mathbf {x}'}\bigg [\sum _{y'\in X} \phi (y', e ',\mathbf {x}') \cdot u_{y'}^{x} \, \bigg ]\varPi ( e , \mathrm {d} e ') \nonumber \\&=\; \sum _{i} p_{i} \, L_i \cdot \Big (\phi ^*(x_i, e ,\mathbf {x}) \mathbb {E}\big [\mathcal {F}_i\big ] + \phi ^*(x_i, e ,\mathbf {x}) - \big (T\phi ^*\big )(x_i, e ,\mathbf {x}) \,\Big ) \nonumber \\&\quad + \; \sum _{x\in X} \pi _x \, n(x,\mathbf {x}) \, L_x \cdot \big (T\phi ^*\big )(x, e ,\mathbf {x}), \end{aligned}$$

where the final equality comes from the definitions of $\mathcal {F}$ and *T*. The per-ploidy reproductive value ($\phi ^*$) has been substituted, so that by the definition of reproductive value we may use $(T\phi ^*) = \phi ^*$, which together with Eq. (12) gives

17 $$\begin{aligned} \sum _{x\in X} \pi _x \, n(x,\mathbf {x}) \, L_x \cdot (T\phi ^*)(x, e ,\mathbf {x}) \;=\; \sum _{x\in X} \pi _x \,n(x,\mathbf {x}) \, L_x \cdot \phi ^*(x, e ,\mathbf {x}) \;=\; {\text {ave}}_{L \phi ^*} p . \end{aligned}$$

Combining (16) and (17) we get

$$\begin{aligned} \mathbb {E}\big [ {\text {ave}}_{\phi ' } {p^*}' \big ] \;&= \; \sum _{i\in I} \, \phi _i \cdot p_i \, \mathbb {E}\big [\mathcal {F}_i\big ] \;+\; {\text {ave}}_{\phi } p , \end{aligned}$$

and, as $$\sum _i{\phi _i }=1$$,

$$\begin{aligned} \mathbb {E}\big [ \varDelta ^* {\text {ave}}_{ \phi } p \big ] \;=\; {\text {ave}}_{\phi } \big (p \, \mathbb {E}\big [\mathcal {F}\big ] \big ) = {\text {cov}}_{\phi }\left( p, \,\mathbb {E}\big [\mathcal {F}\big ]\right) , \end{aligned}$$

where the second equality follows when we recall from Lemma 2 that ${\text {ave}}_{\phi } (\mathcal {F}) =0$, hence ${\text {ave}}_{\phi } (\mathbb {E}\big [\mathcal {F}\big ]) =0$. The equality of the outer terms is the statement of the theorem. $\square$

This first Price Equation holds conditional on the initial condition $( e ,\mathbf {x},\mathbf {g})^{(0)}$ of the Taylor switch stochastic process. It will be useful to find a form in which the Price Equation holds in expectation over the different possible initial conditions.

### Removing dependence on initial conditions

Theorem 2 gives us the expected change in weighted allele frequencies for a given population *I* with environment *e* and class and genotype arrays $(\mathbf {x},\mathbf {g})$ , and a function $p:I\rightarrow \mathbb {R}$ denoting the *p*-scores. In this section we remove that dependency and ask what the expected reproductive-value-weighted change in a *p*-score is *on average* over the initial conditions of the Taylor year. As a preliminary, we re-write the statement of Theorem 2 to highlight the dependencies on *e* and $(\mathbf {x},\mathbf {g})$, which requires us to say what we mean by a *p*-score when the population is not of fixed size or known class-composition. A *p*-score is a weighted sum of allele frequencies, and so we define a *p*-score by weights $\lambda _k$ for each allele *k*. Then, in any population with given individual genotypes $g_i$, we can calculate the *p*-score of individual *i* as $p_i = \sum _k{\lambda _k g_i^k}$. This definition allows us to reach conclusions about mean *p*-scores in a random population drawn from the ergodic distribution of the neutral stochastic process. On this basis, we rewrite Theorem 2 as

18 $$\begin{aligned} \mathbb {E}\big [ \varDelta ^* {\text {ave}}_{\phi } p \,\big |\, e , \mathbf {x}, \mathbf {g}\, \big ]&= {\text {cov}}_{\phi }\left( \, p, \, \mathbb {E}[\mathcal {F}\,\big |\, e , \mathbf {x}, \mathbf {g}\, ]\, \right) . \end{aligned}$$

The next step is to take unconditional expectations on both sides, thus averaging over the ergodic distribution of the neutral stochastic process. This leads to a formula for the expected change in the reproductive-value-weighted frequency of *p* over the set of possible initial conditions.

#### Corollary 1

(The Price Equation—ensemble version) The expected change in the reproductive-value-weighted mean of an arbitrary *p*-score *p*, over the Taylor year, before mutation, is

$$\begin{aligned} \mathbb {E}\big [ \varDelta ^* {\text {ave}}_{\phi } p \, \big ]&= \mathbb {E}\big [ {\text {cov}}_{\phi }\left( \,p, \, \mathbb {E}\big [\mathcal {F}\big |\, e, \mathbf {x}, \mathbf {a}\, \big ] \;\right) \big ]. \end{aligned}$$

Note that the genotype-through-phenotype assumption on $\mathbf {w}$ allows the expectation of $\mathcal {F}$ to be conditioned on $\mathbf {a}$ with exactly the same effect as conditioning on $\mathbf {g}$, which removes an additional genetic element from the statement of the Price Equation. The conditional and unconditional Price Equations will each be employed now to derive a corresponding fundamental theorem of natural selection.

## A generalised fundamental theorem of natural selection

The technical side of Fisher (1930)’s fundamental theorem of natural selection is now well understood (Grafen 2015a), though see Ewens and Lessard (2015) for an alternative view. Here we prove one further version that conditions on the state at the beginning of the Taylor year, and so simply shows that the theorem holds under the uncertainty that unfolds in the course of one generation, and then a further fully stochastic version over the random starting state of the Taylor switch process.

### Theorem 3

(FTNS precursor for one year, with stochastic fitnesses) Suppose the breeding values for expected fitnesses in the Taylor year, conditioning on the initial state, are $\beta _i$, and that these equal the values of some *p*-score *p*. Then the expected change in the mean of *p*, before mutation, equals the variance in *p*. Formally,

19 $$\begin{aligned} \mathbb {E}[\,\varDelta ^* {\text {ave}}_{\phi } p \, | \, e ,\mathbf {x},\mathbf {a}\,] = {\text {var}}_{\phi } p . \end{aligned}$$

Although this is a discrete-time version of the theorem, it lacks the denominator on the right hand side noted by Ewens (1989), which has disappeared because of the requirement here that the total reproductive value remains constant, which in turn causes the mean fitness to be zero and so the denominator to be one.

### Proof

We follow the standard way to move from the Price Equation to the fundamental theorem, by applying the Price Equation to the *p*-score that defines the breeding values of fitness. Here the breeding value is of expected fitness. Let $(\beta _i)_{i\in I}$ be defined as weighted sums of allele frequencies $(\sum _k{\lambda _k g^i_k})_{i\in I}$ that minimise ${\text {ave}}_{\phi } (\mathbb {E}[F_i | e ,\mathbf {x},\mathbf {g},\mathbf {a}] - \beta _i)^2$, where we exceptionally notate the subscript *i* for clarity. Appendix C calculates normal equations on this basis. We begin with the Price Equation in Eq. (18), apply the definition of covariance, eliminating the second term because ${\text {ave}}_{\phi } \mathcal {F} =0$ as noted in Lemma 2, and then apply the normal Eq. (21), to obtain

$$\begin{aligned} \mathbb {E}\big [ \varDelta ^* {\text {ave}}_{\phi } p \, | \, e ,\mathbf {x},\mathbf {a}\,\big ]&\; = \; {\text {cov}}_{\phi }\left( \, p, \, \mathbb {E}[\mathcal {F}| e, \mathbf {x},\mathbf {a}\, ] \;\right) \\&\; = \; {\text {ave}}_{\phi } (\,p\; \mathbb {E}[\mathcal {F}| e, \mathbf {x},\mathbf {a}]\,) - {\text {ave}}_{\phi } (p) {\text {ave}}_{\phi } (\mathbb {E}[\mathcal {F}| e, \mathbf {x},\mathbf {a}\, ]) \\&\; = \; {\text {ave}}_{\phi } (p^2 ) . \end{aligned}$$

Now, the normal Eq. (20) tells us that ${\text {ave}}_{\phi } p =0$, and so the final line equals ${\text {var}}_{\phi } p$, thus completing the proof. $\square$

We now prove an unconditional fundamental theorem, in which the breeding values are the best predictors of fitness over the ensemble of possible years, and not for any one specific year.

### Theorem 4

(FTNS precursor over the stochastic ensemble) Let ensemble breeding values $\beta _i(\mathbf {g}):=\sum _k{\lambda _k g_i^k}$ be defined by the minimisation with respect to $(\lambda _k)_{k\in \mathcal {K}}$ of $\mathbb {E}\big [ {\text {ave}}_{\phi } \big ((\mathbb {E}[\mathcal {F}| e,\mathbf {x},\mathbf {a}\,] - \beta (\mathbf {g}))^2\big ) \big ]$. Let the *p*-score defined by these allelic weights be *p*. Then the expected change in the reproductive-value-weighted mean of *p* over the Taylor year, before mutation, is given by the sum of the ensemble expectation of the population variance of breeding value and the ensemble variance of the population mean breeding value. Formally,

$$\begin{aligned} \mathbb {E}\big [ \varDelta ^* {\text {ave}}_{\phi } p \, \big ] \; = \; \mathbb {E}[{\text {var}}_{\phi } p ] + \mathbb {V}[{\text {ave}}_{\phi } p ]. \end{aligned}$$

### Proof

The argument of the previous proof, but with each term wrapped in an unconditional expectation and with ensemble breeding values and using the normal Eq. (23), takes us up to

$$\begin{aligned} \mathbb {E}\big [ \varDelta ^* {\text {ave}}_{\phi } p \,\big ] \; = \; \mathbb {E}\big [ {\text {ave}}_{\phi } (p^2 ) \big ], \end{aligned}$$

but now ${\text {ave}}_{\phi } (p^2 )$ is a random variable, to which we apply the definition of variance, and then note that $\mathbb {E}[ {\text {ave}}_{\phi } p ]=0$ by the normal Eq. (22), to obtain

$$\begin{aligned} \mathbb {E}\big [ \varDelta ^* {\text {ave}}_{\phi } p \,\big ] \;=\; \mathbb {E}\big [({\text {ave}}_{\phi } p )^2 + {\text {var}}_{\phi } (p) \big ] \; =\; \mathbb {E}[\, {\text {var}}_{\phi } p \,] + \mathbb {V}[ \,{\text {ave}}_{\phi } p \,], \end{aligned}$$

completing the proof. $\square$

### Remark 10

It is hard to imagine a clearer demonstration of the value not only of keeping distinct the population statistical operators from the expectation and variance operators over uncertainty, as recommended by van Veelen (2005), but also of the fact that both kinds of operators are useful in studying stochastic finite populations.

Both theorems are expressed in terms of a *p*-score that equals the $\beta _i$, to avoid the confusion over whether the change in breeding value is between the mean breeding value at $t=0$ and the mean breeding value at $t=1$ (which is not the case), or instead the difference in the mean of breeding value, calculating the $t=1$ breeding values on the basis of the $t=0$ allelic weights (which is the case). As with Fisher’s original version, if any selection takes place, it will alter the phenotypes in the population and change the reproductive values, so that fitness and the breeding values will also change, and the background phenotype $a_0$ will have to be reconsidered.

These two fundamental theorems have different interpretations. The first theorem deals with quantities that are conditional on the initial state of the Taylor switch process. It asserts that some quantity is increased over the Taylor year when averaging over the uncertainty that unfolds during that year, and specifies that quantity with a set of allelic weights. It makes sense that different environments will favour different traits, and so those allelic weights will differ according to the environment. The second, unconditional, theorem asserts the ensemble mean of some quantity increases on average, and specifies a single set of allelic weights for the whole ensemble. Those allelic weights are the breeding values of a notion of fitness that is ‘best on average over the whole set of stochastic environments and population class arrays’. It is, perhaps, surprising that such a quantity exists, ready to inherit the term ‘fitness’, and this demonstration is one major point of fundamental theorems. Here, fitness is discovered in a very general and abstract model, still operating as the property of an individual, and still with at least a sense of being maximised because the expected change in its mean cannot be negative.

## Discussion

We begin by setting bounds on the considerable generality aspired to by our model, by placing it in the wider context of other models of evolution. Reproductive value is a central concept: using it in our model has uncovered two ambiguities in Fisher’s use of the term, and we also discuss replacing the current ‘asymptotic gene pool’ definition with a more here-and-now axiomatic alternative that in many ways makes more sense. The model is part of the formal darwinism project, and we briefly sketch the turning point it represents there. Finally, we consider briefly the implications of our main results.

### Different mathematical approaches to the study of evolution

There are many mathematical literatures that link Mendel’s rules to Darwinian evolution, but they have different emphases. Mathematical population genetics stays very close to the Mendelian machinery (e.g. Ewens 2004), with the work of Charlesworth and colleagues (see Charlesworth 1980, 1994) going furthest towards Darwinian ideas; adaptive dynamics makes necessary assumptions to provide an ecologically useful representation of the effects of natural selection, including extinction and speciation (see Metz et al. 1992, 1996; Dieckmann and Law 1996; Geritz et al. 1998; Durinx et al. 2008; Metz 2012); other literatures such as that of Taylor and colleagues (e.g. Taylor 1990, 1996; Taylor et al. 2007) and that of Rousset and colleagues (e.g. Rousset 2004; Lehmann et al. 2006), aim to make reasonable genetic assumptions to solve important modelling problems suggested by behavioural ecology, and within their frameworks to provide general results about inclusive fitness. There are also literatures (e.g. Hammerstein 1996; Eshel et al. 1998) that aim to investigate how well the theory of evolutionarily stable strategies (Maynard Smith and Price 1973; Maynard Smith 1982) represents the outcome of full genetic models. The formal darwinism project (Grafen 1999, 2002, 2006a, b; Batty et al. 2014) aims to justify the individual-as-maximising agent analogy in terms of population genetics, but eschews dynamic sufficiency in order to obtain results that apply to a wider range of genetical systems. Recent work by Lehmann and Rousset (2014a) and Lehmann et al. (2015) also investigate the individual-as-maximising-agent analogy, within dynamically sufficient models. For clarity, fitness-maximisation is used to mean that a variant phenotype could not spread in the context set by a resident phenotype, and not that the resident phenotype maximises some global function (in the sense of Metz et al. 2008), an important distinction also specifically remarked on technically by, for example, Rousset (2004, pp. 103 and 195), and made non-technically by Dawkins (1980). At the most abstract level, in which biologists ask ‘what *is* biological design?’ or ‘what can be said about the operation of natural selection *in general*?’, two main formalisms have been developed, namely the fundamental theorem of natural selection of Fisher (1930) and the covariance selection mathematics of Price (1970, 1972).

The model of the present paper extends the formal darwinism project, and generalises the fundamental theorem and the Price Equation to fluctuating demography, all the time recognising that this whole approach is only one of many to the mathematical study of evolution. The model lacks the dynamic sufficiency of mathematical population genetics, it assumes a stationary (if Markov) environment and a single species while adaptive dynamics is about longer term evolutionary change, it does not articulate (though does not rule out) interactions between individuals unlike the ESS theories, and it does not permit those interactions to depend on genetic similarity unlike inclusive fitness models.

### Reproductive value

It has not to our knowledge been noted before that Fisher (1930)’s verbal and mathematical conceptions of reproductive value are inconsistent with each other. Fisher describes reproductive value on page 27 as answering the question “To what extent will persons of this age, on the average, contribute to the ancestry of future generations?”. But the contribution to future ancestry of an individual must equal the sum of its genetic share of the contributions of its offspring, as the contribution is made up of those parts, whereas in Fisher’s mathematical definition he discounts offspring contributions by the time of their production, using a discount factor that turns out to equal the population growth rate. Fisher never uses his concept to decide whether a 40 year old in 1930 makes the same contribution to ‘the ancestry of future generations’ as a 40 year old in 1958, but his two definitions would give different answers (except in populations with a zero growth rate). In stochastic models, in which the environment and class-distribution are different in different states of the world next year, the difference between the two definitions also matters as it affects how we average this year over the possible environments and class distributions next year. The simple results obtained above derive from applying Fisher’s verbal definition, by insisting that the total reproductive value of the population is always constant (here taken to be one), and that an individual’s reproductive value *equals* the expected sum of the reproductive values of its descendants next year (including its possibly surviving self). A number of previous authors including Tuljapurkar (1989), Rousset (2004); Rousset and Ronce (2004) also impose a constant sum, and the calculation based on a total reproductive value of 1 is now standardly derived using coalescent theory (Rousset 2004; Rousset and Ronce 2004). This approach converts Fisher (1927)’s pretty demographic result that the population growth rate measured as total reproductive value is always constant even while the population size and structure are not constant, into a dull definitional imposition of a zero growth rate.

A second ambiguity in Fisher’s use of reproductive value is that in the demographic application for the fundamental theorem, he ensures (by assuming the age-schedule of vital rates remains the same, and by applying the discounting mentioned in the previous paragraph) that the per-capita reproductive values of each age remain constant over time, while in his sex ratio argument (Fisher 1930, pp. 158–160) he shows it is the total reproductive values of the classes (females and males) that remain constant. A significant advance in the present stochastic model incorporates both of these possibilities, as it permits density dependence that would alter the age-schedule of vital rates, and its derivation incorporates the underlying logic of Fisher’s argument that total reproductive values of females and males are equal. (Incidentally, it is not widely understood that even under diploidy, this equality holds good in general only with non-overlapping generations; Goodman 1982; Grafen 2014b). Thus, the current paper’s concept unites Fisher’s two uses of reproductive value, though this is not strictly a generalisation as the definition is different from the demographic use, as noted above.

Fisher (1930) and Price and Smith (1972) both worried about the definitions of reproductive value and fitness. One kind of puzzle is reconciling the results with ecology. Fisher (pp. 41–46) constructs his ‘deteriorating environment’ model to try to reconcile the fundamental theorem’s insistence that natural selection increases mean fitness with the ecological reality that mean fitness must be about zero most of the time. The current model has a more sophisticated treatment and, applied to Fisher’s model, allows the age-specific schedule of vital rates to depend also on the population size and age-structure, and on the environment. This in turn allows density-dependence to operate, with population growth and shrinkage when the population is small and large, respectively, without any genetic changes having to take place. The second kind of worry is whether the results hold when natural selection is changing the genotypes and phenotypes of the population. The issue, both for Fisher and the present paper, is that the genetic changes alter the basic parameters on which reproductive value is calculated. In Fisher’s case these are the whole age-schedule of vital rates, and here the background phenotype $a_0$ in the neutral stochastic process. Fisher’s ‘deteriorating environment’ model attempts to elevate the viewpoint, and study how natural selection’s improvement of the population is balanced by the changes to the species’ environment. Price and Smith’s whole paper is about how to define reproductive value, and assumes that it should in principle be measured in a ‘real’ population that is subject to natural selection and other changes; they then say they cannot see how to implement this principle, and resort to a ‘null hypothesis’ evaluation *faute de mieux*. We regard the principled position as completely unimplementable, as it would require knowing the whole evolutionary future of the species: it is unhelpful to decide that reproductive value cannot be usefully modelled in a species whose lineage eventually becomes extinct (the fate of *all* lineages), and even on a shorter term view we would need to decide which daughter to follow after speciation (following more than one would create problems of non-uniquess of reproductive value). Over such a longer term, the environment will be expected to change radically and non-stationarily, rendering that future irrelevant to evolution today. Thus, we employ the ‘Taylor switch’ as a version of the ‘null hypothesis’ approach that suits our needs. The illogicality of the asymptotic view when taken to extremes, together with the fact that reproductive value seems to do something sensible in our models, suggests there may be an alternative conceptual framework that represents our real purpose, for which the asymptotic calculation is merely one implementation.

Such an alternative logical basis for reproductive value is employed by Grafen (2015b), in a deterministic model. This approach takes one well-known property of reproductive value, and essentially turns it into the definition of reproductive value, but through a condition on fitnesses. The well-known property is that reproductive-value weighting of frequencies implies that neutral genes do not change in their frequencies (e.g. Taylor 1990; Rousset 2004; Rousset and Ronce 2004). The ‘neutrality condition’ on fitnesses is that the additive genetic variance in fitness must be exactly zero, whenever all of the genotypic variability currently in the population is currently neutral, in the sense that differences between genotypes do not cause differences between phenotypes. In these circumstances, natural selection cannot be operating, as it requires variability in genotypes to bring about variability in phenotypes that in turn changes gene frequencies. This switch of logical basis articulates Grafen (2015a)’s claim that the real value in the fundamental theorem lies not in calculations about some hypothetical distant gene pool, but rather in detecting natural selection in the present. Indeed, if the fundamental theorem is regarded as essentially defining natural selection, then the name ‘fundamental theorem of natural selection’ seems very appropriate. The current stochastic model still uses the asymptotic gene pool basis of reproductive value, but a refoundation may be possible.

Choosing reproductive value so as to define how much of evolutionary change is ‘due to natural selection’ is, contrary perhaps to first impressions, an important way to choose. If we believe Darwin’s characterisation of natural selection as an improving process, it is reasonable to ask of a Mendelian model ‘what does improvement mean?’, and to hope for a definition of what exactly is improved. Clearly, the improvement must be understood as ‘improvement caused by natural selection, even though other forces may lead to a net deterioration’, and on average for a species the net improvement is likely to be zero on average. This point was already clear to Fisher (1930, pp. 41–46). This isolation of the effect of natural selection was characterised by Price (1972) as a partial change in mean fitness. Grafen (2015a) claims that the point of the fundamental theorem is the combination of the definition of fitness and the proof that the effect of natural selection is always to improve it. Pursuing this philosophy also led to the handling of mutation in the present model by looking at changes over the cycle of genetic events from just after one year’s mutation events to just before the next year’s; the incompleteness is notationally marked by using $\varDelta ^*$ rather than plain $\varDelta$. This simple exclusion avoids the complications, studied by Tarnita and Taylor (2014), that arise in overlapping generations because mutation occurs in newborns but not in adults, which can lead to mutation affecting different classes differently. To undertake an exact description of the changes in genotype frequencies, these complications must of course be included, but we regard them as not always necessary when a study is focussed on natural selection.

Thus, the biological viewpoint that we wish to understand the effects of natural selection has suggested a modification to the straightforward ‘dynamical systems’ approach of studying equilibria in population genetic models. The Price Equation (Price 1970, 1972) can be regarded as a generalisation of the secondary theorem of natural selection of Robertson (1966, 1968): both share a wish with Fisher (1930, pp. 36–37) to draw conclusions about observables without denying the underlying genetics, which is regarded as unobservable. Like the fundamental theorem, they both hold without considering how gametes combine to form individuals, and so by themselves lack the capacity to track exact genotype frequencies through time (though see Frank 1998, for an argument that the Price Equation can.). We have just noted the point that the change in mean fitness calculated in Fisher (1930)’s fundamental theorem of natural selection is a partial change: specifically, that part due to natural selection. There are two approaches to this focus of the Price Equation and the fundamental theorem on the effects of natural selection. One is to regard the true aim as predicting the full change in mean fitness and the complete change in each trait mean, which requires articulating or abolishing-by-assumption the other causes. The fullest treatment of the fundamental theorem in this ‘completing’ vein is probably Nagylaki (1993), and see also the discussions by Bürger (2000, Chapter III, section 6) and Ewens (2004), on the ‘Mean Fitness Increase Theorem’. The approach taken here, most explicitly in the axiomatic refoundation of reproductive value advocated above, is to regard the partial nature of these evolutionary results as very useful for the study of natural selection, specifically isolating its effects from changes due to other genetic and environmental causes in Mendelian systems: we develop more fully what we regard as Fisher’s philosophy to extend it to the stochastic case. The most immediate use is establishing a fundamental theorem that provides a formal version of the claim that natural selection is an improving process. Our conceptual focus is useful for understanding biological adaptation, and is not intended for the prediction of genotype frequencies.

A number of these points about reproductive value will be returned to in the rest of the discussion.

### In the context of formal darwinism

The formal darwinism project has core papers (Grafen 1999, 2000, 2002, 2006a, b; Gardner and Grafen 2009; Batty et al. 2014; Grafen 2015b) and an interpretative penumbra (Grafen 2007c, 2014a), along with two applications (Grafen 2007a, b) and discussions (Birch 2014; Bourke 2014; Ewens 2014; Gardner 2014; Haig 2014; Lehmann and Rousset 2014a; Okasha and Paternotte 2014; Sarkar 2014; Shelton and Michod 2014). The current paper continues the core development, extending the philosophy to arbitrary classes, Markov environments and fluctuating demography. It simplifies the mathematical development considerably by having a finite population.

Compared to the other core papers, and in line with Grafen (2015b), the current paper has a Price Equation as usual (Theorem 2 and Corollary 1) but also a fundamental theorem (Theorems 3 and 4), but does not move on to explicit fitness-maximisation results that link the Price Equation to optimisation programs under further explicit genetic assumptions. Not only is the construction of the Price Equation arduous enough for one paper, but the generality of the recent results leads to hope for a more direct argument about fitness-maximisation that does not involve specialising to particular genetic architectures. In fact, one main purpose of the explicit fitness-maximising results was to establish that the fitness measure deserved that name: in the more recent work, the surprising simplicity of the results goes a considerable way to showing that this measure of fitness is the right one. Previously, the measure was constructed ad hoc, and then justified by the explicit results. Here, the measure is very naturally constructed on reproductive value. Further work is needed to explore the possibility that these two connections are enough. A likely direction is to follow Grafen (2015b) even more closely, and replace our definition of reproductive value in line with the suggestion in the preceding part of the discussion, which would regard the fundamental theorem as defining natural selection, and confirm that our measure of fitness is appropriate and essentially unique.

The current paper improves in its fitness definition on previous models that incorporated classes, namely Grafen (2006b) and Batty et al. (2014), which effectively used Williams’ reproductive value as fitness (Grafen 2015b, reconciles the two approaches), and differs from both by having a finite population. Batty et al. (2014) does have an explicit treatment of fitness-optimisation, which has the advantage of articulating the strategic (including informational) situation of the individual organism regarded as a maximising agent. It shows that natural selection should lead to organisms having a correct prior distribution over uncertainty, and reacting in a Bayesianly optimal way to whatever information they possess.

A more detailed point is that Grafen (2015b) has a cliff-hanger quality. It greatly simplifies and extends formal darwinism by employing Fisher’s definition of fitness (as articulated by Grafen 2015a) and applying it to an arbitrary class structure, but ends up by providing two Price Equations and two fundamental theorems, which was unsatisfactory for a project that aims to show individuals acting as maximising agents, and which hopes for a unique maximand. The cause of the split was the ambiguity in whether per-capita or total-class reproductive values should be regarded as remaining constant over time. As explained in the preceding part of the discussion, the stochastic version of reproductive value embraces both of those possibilities, and so the current paper re-unifies the threatened split in the formal darwinism project. The refounding of stochastic reproductive value on the axiomatic basis mentioned above would fully combine (Grafen 2015b) with the present paper. That version of the project would still await interactions between relatives (dealt with more primitively in Grafen 2006a) for a putatively final formulation.

One point for further consideration within formal darwinism is the difference between the contributions of the conditional and the ensemble results. The conditional and ensemble Price Equations in Theorem 2 and Corollary 1 are simply related within the model. A difference may lie in trying to apply them to a real situation, when we need to ask what background phenotype value $a_0$ to choose to work with. In the fundamental Theorems 3 and 4, although fitness is the same, we have an ensemble breeding value of fitness for the whole process, and a conditional breeding value of fitness for each configuration of a year, and generically these are all different. In each case, a more detailed consideration should show for which purposes the shorter-term result is relevant, and for which purposes the longer-term result should be preferred. Applications to model systems may be the best way forward.

### Interpretations of the main results

The significance of Price Equations and fundamental theorems in deterministic settings is still unsettled (Ewens 2004; Edwards 2014; Grafen 2015a; Ewens and Lessard 2015), and no attempt at a final account can be offered here. Here, we note some uncontroversial technical starting points for considering the stochastic versions, and some general remarks. First, the results of the present paper hold with great generality so far as the genetics of the population is concerned. The standard generalities (Price 1970; Ewens 1989) are arbitrary numbers of loci, each with arbitrary numbers of alleles, no restrictions on dominance or epistasis, and arbitrary linkage maps and linkage disequilibrium. Phenotypes depend on genotypes and class in an arbitrary way, and no assumptions are made about the mating system. Here, we also added arbitrary ploidies for each class in an arbitrary class-structure for the population, as well as proving results that hold for any given class-distribution, and that hold with a class distribution that fluctuates over time in response to environmental uncertainty, which itself follows an arbitrary Markov process. One important restriction is that all the loci are assumed to belong to the same coreplicon (Cosmides and Tooby 1981).

A significant caution is that while the results hold exactly, the use of the ‘Taylor switch’ device implies the results hold exactly when there is only one year in which the genotype of an individual can influence its phenotype (although the knock-on effects of that one year may be felt in gene frequencies for ever after), and thus these generalities may not be entirely straightforward to interpret (though for some comfort in that direction, see Grafen 2000; Metz and de Kovel 2013). The lack of dynamic sufficiency is discussed in Sect. 2, and the isolation of the effect of natural selection from other causes is discussed above in Sect. 5.2. One positive feature is that the assumptions are very general, and so the simplicity of the results may suggest new ways of measuring fitness in natural populations.

Now consider the content of our main results. Theorem 1 links reproductive value to martingale theory, as a formal demonstration that reproductive value does represent the ‘fraction of an asymptotic gene pool’. In the context of the alternative foundation of reproductive value, this may seem backwards looking, but it has the merit of setting out exactly what a refoundation has to match in logical terms. The original Price Equation (Price 1970) holds for an arbitrary weighted sum of allele frequencies, and shows that the expected change in the population mean of the weighted sum equals the covariance of that sum with individual fitness, which is regarded as ‘the change due to natural selection’, plus a second ‘property-change’ term (Price 1970; Frank 2012). Here, two further results are proved. First, Theorem 2 conditions on initial conditions for a year, and is very similar to the deterministic class-based result, in which fitness and the change in mean *p*-score are replaced with their expected values, and the second right hand term disappears, because we assume unbiassed transmission and take expectations over Mendelian segregation. Thus, so far as identifying ‘the change due to natural selection’ is concerned, essentially the same result holds in the much more complex setting. Corollary 1 takes an expectation over the ensemble of possible initial conditions for the population, and shows that the expected change in a mean *p*-score equals the unconditional expectation of the covariance of *p*-score with the conditional expectation of fitness, on environment, class and phenotype arrays. The Price Equations have corresponding fundamental Theorems 3 and 4, which show that the expected change in fitness brought about by natural selection is non-negative, a formal embodiment of the idea that natural selection is an improving process, and which can hold good only for very special definitions of fitness while still simultaneously satisfying the neutrality condition of Grafen (2015b).

One major conclusion is that Fisher’s ingenious definition of fitness and his basic approach to combining classes of descendant by reproductive values are very productively applied to the stochastic setting with fluctuating demography. In this setting, we provide a definition of fitness at the individual level under which the fundamental theorem is able to show that natural selection is on average an improving process, and under which the Price Equation can show that natural selection on all traits operates through the expected value of fitness. This goes far beyond mere consistency, but can be regarded as following Fisher’s program to found Darwinism on Mendelism. The ease of extension and simplicity of the results show that Fisher’s logic cuts to the core of the connection between genetics and adaptation.

One major conceptual point concerns the effect of uncertainty on selection. Our Price Equations show that a straightfoward arithmetic average of fitness, over uncertainty of all kinds, predicts the expected effect of selection, in contrast to the common biological theme that the variability of fitness also plays a role (for a general discussion, see Seger and Brockmann 1987). Of course, the discrepancy arises because the definitions of fitness are different. Ours certainly has one advantage, of being defined in the same way over a wide range of situations. More substantively, it is desirable to reanalyse models of bet-hedging in terms of the present approach, to see how the new structure re-interprets those models, and to ask whether the linear-fitness-expectation approach outlined here makes as good, or perhaps even better, sense of the biological phenomena being modelled than the risk-avoiding interpretations.

We conclude by emphasising the major conceptual points at stake. In many whole-organism studies, it is simply assumed that natural selection leads individuals to act as though maximising an individually based quantity called fitness. As partial reassurance that this is a reasonable position to take, the first major point is our Price Equations show that the same formula for expected change applies to all *p*-scores, and so all loci are subjected to the same selection pressure, and we should expect no intra-genomic conflict within a coreplicon. The second is the expected change in a *p*-score is equal to its expected covariance with an individual based measure of fitness, and therefore we expect all tissues within an organism to be selected to dovetail towards individual adaptation. Third, our fundamental theorems show that in each specific set of circumstances, and also averaging over possible circumstances, natural selection acts to improve mean fitness on average. These major points, already established for deterministic cases, are extended in the current work to environmental and class-distribution stochasticity, at the cost of an appropriate definition of reproductive value, and replacing some terms with their expected values. It is the extension to stochasticity not the biological meaning of the results that is new here. These questions are not the usual concern of the dynamically sufficient models of mathematical population genetics, but they underlie the everyday practice of many whole-organism biologists. Our results, therefore, significantly extend, but by no means complete, the articulation of Darwinian ‘improvement’ and ‘fitness’ in terms of Mendelian genetics.

## Proof of Lemma 1

Lemma 1 states that the set $\mathcal {Z}$ is non-empty and *T*-invariant.

### Proof

For $\mathcal {Z}$ non-empty, choose an arbitrary measurable function $g:X\times E\rightarrow (\alpha , \beta )\subset (0,\infty )$ and define $f: X\times E\times \tilde{X}\rightarrow [0,\infty )$ by

$$\begin{aligned} f(x, e ,\mathbf {x}) = \left\{ \begin{array}{cl} \displaystyle {\frac{g(x, e )}{\sum _{y\in X} g(y, e ) \cdot L_y \, n(y,\mathbf {x}) }} \qquad &{} n(x,\mathbf {x}) \ge 1,\\ 0 &{} n(x,\mathbf {x}) = 0. \end{array} \right. \end{aligned}$$

Then *f* is non-negative, bounded by 1, and

$$\begin{aligned} \sum _{x\in X} f(x, e ,\mathbf {v}) \cdot L_x \,n(x,\mathbf {x}) \;=\; \frac{\sum _{x\in X} g(x, e ) \cdot L_x \, n(x,\mathbf {x})}{\sum _{y\in X} g(y, e ) \cdot L_y \, n(y,\mathbf {x})} \;=\; 1 \end{aligned}$$

for all $e \in E$ and $\mathbf {x}\in \tilde{X}$. Hence $f\in \mathcal {Z}$ and $\mathcal {Z}$ is non-empty.

For *T*-invariance, let $f\in \mathcal {Z}$. We have already observed, in establishing that *T* is a well defined linear operator, that $(Tf)\in BM$, and (*Tf*) is non-negative since $f \ge 0$. Fix $( e ,\mathbf {x})\in E\times \tilde{X}$.

$$\begin{aligned}&\sum _{x\in X} n(x,\mathbf {x}) \, L_x \cdot \big (Tf\big )(x, e ,\mathbf {x})\\&\quad =\sum _{x\in X} n(x,\mathbf {x}) \, L_x \cdot \int _E \mathbb {E}_{\mathbf {x}'}\Big [ \sum _{y'\in X} f(y', e ',\mathbf {x}'( e ,\mathbf {x})) \cdot L_{y'} \, u_{y'}^{x} \, \Big ] \, \varPi (e,\mathrm {d} e ') \\&\quad =\int _E \mathbb {E}_{\mathbf {x}'}\Big [ \sum _{y'\in X} f(y', e ',\mathbf {x}'( e ,\mathbf {x})) \cdot L_{y'} \cdot \sum _{x\in X} \big ( n(x,\mathbf {x}) \, L_x \, u_{y'}^{x} \, \big )\Big ] \, \varPi (e,\mathrm {d} e ') \\&\quad =\int _E \mathbb {E}_{\mathbf {x}'}\Big [\sum _{y'\in X} f(y', e ',\mathbf {x}'( e ,\mathbf {x})) \cdot L_{y'} \, n(y',\mathbf {x}'( e ,\mathbf {x})) \, \Big ] \, \varPi ( e ,\mathrm {d} e ') \\&\quad =\int _E \mathbb {E}_{\mathbf {x}'}\big [1\big ] \, \varPi ( e ,\mathrm {d} e ') \\&\quad =1, \end{aligned}$$

where the third line is by Definition 6 of $\mathbf {x}'(e,\mathbf {x})$, and the fourth by Definition 7 of $\mathcal {Z}$. Hence $(Tf)\in \mathcal {Z}$ as required. $\square$

## Existence and uniqueness of reproductive value

### Existence

While reproductive value as defined in Sect. 2 may not always exist in general, it is often possible to arrive at a reproductive value function computationally as the solution to a system of linear equations. We present in this section an abstract existence result for the simplest case of a bounded haploid population on a finite class space with a finite number of possible environments. Existence and uniqueness for a similar definition of reproductive value is discussed for various examples by Grafen (2006b).

Let $X := \{1,2,\ldots ,n\}$ be a class space, $E := \{1,2,\ldots ,m\}$ a set of environments and suppose there is a maximum possible population size *K*.

#### Proposition 1

In this case, there exists a reproductive value function $\phi ^*: X\times E\times \tilde{X}\rightarrow [0,1]$ such that $T\phi ^* = \phi ^*$.

#### Proof

The set of population arrays $\tilde{X}=\bigcup _{k=1,2\ldots K} X^k$ is now finite, and thus so is the Cartesian product $X\times E\times \tilde{X}$. The fixed point space $\mathcal {Z}$ is a subset of functions on $X\times E\times \tilde{X}$, and is clearly convex. The set of functions bounded by 1 on the now finite $X\times E\times \tilde{X}$ may be identified with a closed ball in Euclidean space, and it follows that $\mathcal {Z}$ is closed and compact. The *T*-invariance of $\mathcal {Z}$ is proved in Lemma 1. Hence *T* is continuous from a compact, convex subset of a Euclidean space to itself, so by Brouwer’s fixed point theorem, *T* has a fixed point $\phi ^*$ in $\mathcal {Z}$. Thus we have shown that there exists a reproductive value function $\phi ^*: X\times E\times \tilde{X}\rightarrow [0,1]$ such that $T\phi ^* = \phi ^*$. $\square$

While this is merely a non-constructive proof of existence, in practice, finding the fixed point of a linear operator amounts to solving a system of linear equations and can often be easily implemented in software.

### Uniqueness

Neither Brouwer’s fixed point theorem as used in the example above, nor the definition of reproductive value assert the uniqueness of the fixed point. It should be noted that non-uniqueness also occurs for previous definitions (such as in Grafen 2006b or Engen et al. 2009) of reproductive value as a normalised left eigenvector of some population transition matrix in the case where the eigenspace has dimension ${>}1$.

Suppose that $\phi ^*_1$ and $\phi ^*_2$ satisfy the definition of reproductive value functions. It is easily seen that the convex combination $t\phi ^*_1 + (1-t)\phi ^*_2$ is also a reproductive value function for all $t\in (0,1)$ so the set of reproductive value functions is a convex subset of $\mathcal {Z}$. One example where we should expect such behaviour is when the class space is partitioned into sets $X = X_1 \cup \ldots \cup X_n$ and individuals in a set $X_k$ have offspring only in the same set (perhaps class represents geographical separation). In this case the proportion of the total reproductive value contained in each $X_k$ remains fixed over time but any initial distribution of the total reproductive value over the partition is equally valid. In other words, for any $\alpha _1,\ldots ,\alpha _n\in [0,1]$ such that $\sum _{k=1}^n \alpha _k =1$, if $\phi ^*$ is a reproductive value then so is the function $\phi ^*_\alpha$ defined for $x\in X_k$ by

$$\begin{aligned} \phi ^*_\alpha (x, e ,\mathbf {x}) := \frac{\alpha (x) \cdot \phi ^*(x, e ,\mathbf {x})}{\sum _{y\in X_k} n(y,\mathbf {x}) \cdot L_y\cdot \alpha (y)\cdot \phi ^*(y, e ,\mathbf {x})}, \end{aligned}$$

where understand $\alpha (y)=\alpha _k$ when $y\in X_k$. Non-uniqueness of reproductive value does not affect any of the results or conclusions above. The reproductive-value-weighted *p*-score changes according to Eq. (14) for any function $\phi ^*$ satisfying Definition 5.

## Ensemble breeding values

Calculation of breeding values in a single defined population is a well-known procedure (Falconer 1981). In the text, breeding values are defined over a population with stochastic fitnesses, and also over an ensemble of populations and environments, and here we spell out the rather simple extensions required. Each combination $( e ,\mathbf {x},\mathbf {g})$ can be regarded as defining a population. Individual *i*’s frequency of allele *k* is $g_i^k$, where the sum of $g_i^k$ over the alleles at a single locus equals 1 in each individual, and the breeding value will be defined by allelic weights $(\lambda _k)_{k\in \mathcal {K}}$, and denoted $\beta _i:=\sum _k{\lambda _k g^i_k}$. This appendix presents normal equations for use in the main text, and we note that the quantity to be minimised must also be weighted by reproductive value, if the breeding values are to fulfil their role in the argument.

The breeding values for a population simply replace fitness with expected fitness. For a deterministic derivation with reproductive-value weighting, see Grafen (2015b). The normal equations for the regression defining breeding value are then

20 $$\begin{aligned} {\text {ave}}_{\phi } \big (\mathbb {E}[\mathcal {F}| e,\mathbf {x},\mathbf {a}\,] - \beta )\big ) =&0 \end{aligned}$$

21 $$\begin{aligned} {\text {ave}}_{\phi } \big (\beta \cdot (\mathbb {E}[\mathcal {F}| e,\mathbf {x},\mathbf {a}\,] - \beta )\big ) =&0 \end{aligned}$$

The ensemble breeding values will be elaborated further. It is convenient that the use of allelic weights allows us to define breeding values in any given type of year, even though the population varies, including in size, over the ensemble. We will indicate the dependence of the allelic weights on the genotype array by writing $\beta _i=\beta _i(\mathbf {g})$, partly to emphasise that it does not depend on any of the other variables. The quantity to be minimised by choice of allelic weights $\lambda _k$ is the expectation over the ensemble of the population reproductive-value-weighted mean squared deviation of individual fitness from breeding value, formally

$$\begin{aligned} \mathbb {E}\big [ {\text {ave}}_{\phi } \big (\;(\mathbb {E}[\mathcal {F}| e,\mathbf {x},\mathbf {a}\,] - \beta (\mathbf {g}))^2\;\big ) \big ]. \end{aligned}$$

Substituting $\sum _k{\lambda _k g_i^k}$ for $\beta (\mathbf {g})$ gives

$$\begin{aligned} A := \mathbb {E}\bigg [ {\text {ave}}_{\phi } \bigg ( \;\Big (\mathbb {E}[\mathcal {F}| e,\mathbf {x},\mathbf {a}\,] - \sum _k{\lambda _k g_i^k}\Big )^2\; \bigg ) \bigg ], \end{aligned}$$

and differentiating *A* with respect to $\lambda _k$ gives

$$\begin{aligned} A_k := \mathbb {E}\bigg [ {\text {ave}}_{\phi } \Big ( -2 g_i^k \Big ( \mathbb {E}[\mathcal {F}| e,\mathbf {x},\mathbf {a}\,] - \sum _k{\lambda _k g_i^k} \Big ) \Big ) \bigg ]. \end{aligned}$$

Setting these derivatives to zero sufficiently defines the minimising values $\lambda ^*_k$. Now letting $n_L$ denote the number of loci, so that $\sum _k{g_i^k}=n_L$ for each individual, we obtain the normal equations by forming sums as follows,

$$\begin{aligned} 0 = \frac{-1}{2 n_L} \sum _k{A_k}&= \mathbb {E}\bigg [ {\text {ave}}_{\phi } \Big ( \; \mathbb {E}[\mathcal {F}| e,\mathbf {x},\mathbf {a}\,] - \sum _k{\lambda ^*_k g_i^k} \; \Big ) \bigg ] \\ 0 = - \sum _k{\lambda ^*_k A_k}&= \mathbb {E}\bigg [ {\text {ave}}_{\phi } \bigg (\Big (\sum _k{\lambda ^*_k g_i^k}\Big )\cdot \Big (\mathbb {E}[\mathcal {F}| e,\mathbf {x},\mathbf {a}\,] - \sum _k{\lambda ^*_k g_i^k}\Big ) \bigg ) \bigg ]. \end{aligned}$$

We now complete the work of the appendix as we write the normal equations by substituting in $\beta _i(\mathbf {g})$ as appropriate, returning to our usual convention of suppressing the individual subscript *i* inside population statistical operators, and suppressing the notation for the dependence of $\beta _i$ on $\mathbf {g}$:

22 $$\begin{aligned} \mathbb {E}\Big [ {\text {ave}}_{\phi } \big (\mathbb {E}[\mathcal {F}| e,\mathbf {x},\mathbf {a}\,] - \beta \big ) \Big ]&= 0 \end{aligned}$$

23 $$\begin{aligned} \mathbb {E}\Big [ {\text {ave}}_{\phi } \Big (\beta \cdot \big (\mathbb {E}[\mathcal {F}| e,\mathbf {x},\mathbf {a}\,] -\beta \big )\Big ) \Big ]&= 0. \end{aligned}$$

## References

[CR1] Barton NH, Etheridge AM (2011). The relation between reproductive value and genetic contribution. Genetics.

[CR2] Batty CJK, Crewe P, Grafen A, Gratwick R (2014). Foundations of a mathematical theory of darwinism. J Math Biol.

[CR3] Birch J (2014). Has grafen formalized darwin?. Biol Philos.

[CR4] Bourke AFG (2014). The gene’s-eye view, major transitions and the formal darwinism project. Biol Philos.

[CR5] Bürger R (2000). The mathematical theory of selection, recombination and mutation.

[CR6] Charlesworth B, Markl H (1980). Models of kin selection. Evolution of social behaviour: hypotheses and empirical tests.

[CR7] Charlesworth B (1994). Evolution in age-structured populations.

[CR8] Cosmides LM, Tooby J (1981). Cytoplasmic inheritance and intragenomic conflict. J Theor Biol.

[CR9] Coulson T, Tuljapurkar S, Childs DZ (2010). Using evolutionary demography to link life history theory, quantitative genetics and population ecology. J Anim Ecol.

[CR10] Dawkins R, Barlow G, Silverberg J (1980). Good strategy or evolutionarily stable strategy. Sociobiology: beyond nature/nurture?.

[CR11] Dieckmann U, Law R (1996). The dynamical theory of coevolution: a derivation from stochastic ecological processes. J Math Biol.

[CR12] Durinx M, Metz JAJ, Meszéna G (2008). Adaptive dynamics for physiologically structured population models. J Math Biol.

[CR13] Edwards AWF (2014). R.A. Fisher’s gene-centred view of evolution and the fundamental theorem of natural selection. Biol Rev.

[CR14] Engen S, Lande R, Saether B-E (2009). Reproductive value and fluctuating selection in an age-structured population. Genetics.

[CR15] Eshel I, Feldman MWF, Bergman A (1998). Long-term evolution, short-term evolution and population genetics. J Theor Biol.

[CR16] Ewens WJ (1989). An interpretation and proof of the fundamental theorem of natural selection. Theor Popul Biol.

[CR17] Ewens WJ (2004). Mathematical population genetics I. Theoretical introduction.

[CR18] Ewens WJ (2014). Grafen, the Price equations, fitness maximization, optimisation and the fundamental theorem of natural selection. Biol Philos.

[CR19] Ewens WJ, Lessard S (2015). On the interpretation and relevance of the fundamental theorem of natural selection. Theor Popul Biol.

[CR20] Falconer DS (1981). Introduction to quantitative genetics.

[CR21] Fisher RA (1927). The actuarial treatment of official birth records. Eugen Rev.

[CR22] Fisher RA (1930) The genetical theory of natural selection. Oxford University Press, Oxford **(See Fisher (1999) for a version in print)**

[CR23] Fisher RA (1999) The genetical theory of natural selection. Oxford University Press, Oxford, 1999. Variorum Edition of 1930 OUP edition and 1958 Dover edition, edited by J. Henry Bennett

[CR24] Frank S A (1998). The foundations of social evolution.

[CR25] Frank S A, Svensson E, Calsbeek R (2012). Wright’s adaptive landscape versus Fisher’s fundamental theorem. The adaptive landscape in evolutionary biology.

[CR26] Gardner A (2014). Life, the universe and everything. Biol Philos.

[CR27] Gardner A, Grafen A (2009). Capturing the superorganism: a formal theory of group adaptation. J Evol Biol.

[CR28] Geritz SAH, Kisdi E, Meszéna G, Metz JAJ (1998). Evolutionarily singular strategies and the adaptive growth and branching of the evolutionary tree. Evol Ecol.

[CR29] Goodman D (1982). Optimal life histories, optimal notation, and the value of reproductive value. Am Nat.

[CR30] Grafen A (1999). Formal Darwinism, the individual-as-maximising-agent analogy, and bet-hedging. Proc R Soc Ser B.

[CR31] Grafen A (2000). Developments of Price’s equation and natural selection under uncertainty. Proc R Soc Ser B.

[CR32] Grafen A (2002). A first formal link between the Price equation and an optimisation program. J Theor Biol.

[CR33] Grafen A (2006). Optimisation of inclusive fitness. J Theor Biol.

[CR34] Grafen A (2006). A theory of Fisher’s reproductive value. J Math Biol.

[CR35] Grafen A (2007). Inclusive fitness on a cyclical network. J Evol Biol.

[CR36] Grafen A (2007). Detecting kin selection at work using inclusive fitness. Proc R Soc Ser B.

[CR37] Grafen A (2007). The formal Darwinism project: a mid-term report. J Evol Biol.

[CR38] Grafen A (2008). The simplest formal argument for fitness optimization. J Genet.

[CR39] Grafen A (2014). The formal darwinism project in outline. Biol Philos.

[CR40] Grafen A (2014). Total reproductive value for females and for males in sexual diploids are not equal. J Theor Biol.

[CR41] Grafen A (2015). Biological fitness and the fundamental theorem of natural selection. Am Nat.

[CR42] Grafen A (2015). Biological fitness and the Price Equation in class-structured populations. J Theor Biol.

[CR43] Haig D (2014). Genetic dissent and individual compromise. Biol Philos.

[CR44] Hammerstein P (1996). Darwinian adaptation, population-genetics and the streetcar theory of evolution. J Math Biol.

[CR45] Lehmann L, Rousset F (2014). Fitness, inclusive fitness and optimization. Biol Philos.

[CR46] Lehmann L, Rousset F (2014). The genetical theory of social behaviour. Philos Trans R Soc B.

[CR47] Lehmann L, Perrin N, Rousset F (2006). Population demography and the evolution of helping behaviors. Evolution.

[CR48] Lehmann L, Alger I, Weibull J (2015) Does evolution lead to maximizing behavior? Evolution 69:1858–1873 10.1111/evo.12701 26082379

[CR49] Lewontin RC (1974). The genetic basis of evolutionary change.

[CR50] Maynard Smith J (1982). Evolution and the theory of games.

[CR51] Maynard Smith J, Price GR (1973). The logic of animal conflict. Nature.

[CR52] Metz JAJ, Hastings A, Gross LJ (2012). Adaptive dynamics. Encyclopedia of theoretical ecology.

[CR53] Metz JAJ, de Kovel CGF (2013). The canonical equation of adaptive dynamics for mendelian diploids and haplo-diploids. Interface Focus.

[CR54] Metz JAJ, Nisbet RM, Geritz SAH (1992). How should we define “fitness” for general ecological scenarios?. Trends Ecol Evol.

[CR55] Metz JAJ, Geritz SAH, Meszéna G, Jacobs FJA, van Heerwaarden JS, van Strien S, Lunel SV (1996). Adaptive dynamics, a geometrical study of the consequences of nearly faithful reproduction. Stochastic and spatial structures of dynamical systems.

[CR56] Metz JAJ, Mylius SD, Dieckmann O (2008). When does evolution optimize?. Evol Ecol Res.

[CR57] Nagylaki T (1993). The evolution of multilocus systems under weak selection. Genetics.

[CR58] Okasha S, Paternotte C (2014). Adaptation, fitness and selection-optimality links. Biol Philos.

[CR59] Price GR (1970). Selection and covariance. Nature.

[CR60] Price GR (1972). Extension of covariance selection mathematics. Ann Hum Genet.

[CR61] Price GR, Smith CAB (1972). Fisher’s Malthusian parameter and reproductive value. Ann Hum Genet.

[CR62] Rice S (2008). A stochastic version of the Price equation reveals the interplay of deterministic and stochastic processes in evolution. BMC Evol Biol.

[CR63] Robertson A (1966). A mathematical model of the culling process in dairy cattle. Anim Prod.

[CR64] Robertson A, Lewontin RC (1968). The spectrum of genetic variation. Population biology and evolution.

[CR65] Rosenblatt M (1971). Markov processes. Structure and asymptotic behavior.

[CR66] Rousset F (2004). Genetic structure and selection in subdivided populations.

[CR67] Rousset F, Ronce O (2004). Inclusive fitness for traits affecting metapopulation demography. Theor Popul Biol.

[CR68] Sarkar S (2014). Formal darwinism. Biol Philos.

[CR69] Schechter E (1997). Handbook of analysis and its foundations.

[CR70] Seger J, Brockmann HJ (1987). What is bet-hedging?. Oxf Surv Evolut Biol.

[CR71] Shelton DE, Michod RE (2014). Levels of selection and the formal darwinism project. Biol Philos.

[CR72] Tarnita CE, Taylor PD (2014). Measures of relative fitness of social behaviors in finite structured population models. Am Nat.

[CR73] Taylor PD (1990). Allele-frequency change in a class-structured population. Am Nat.

[CR74] Taylor PD (1996). Inclusive fitness arguments in genetic models of behaviour. J Math Biol.

[CR75] Taylor PD, Day T, Wild G (2007). Evolution of cooperation in a finite homogeneous graph. Nature.

[CR76] Tuljapurkar S (1989). An uncertain life: demography in random environments. Theor Popul Biol.

[CR77] Tuljapurkar S (1990). Population dynamics in variable environments.

[CR78] van Veelen M (2005). On the use of the Price equation. J Theor Biol.

[CR79] Williams GC (1966). Natural selection, the costs of reproduction, and a refinement of Lack’s principle. Am Nat.

